# Evaluation of rate law approximations in bottom-up kinetic models of metabolism

**DOI:** 10.1186/s12918-016-0283-2

**Published:** 2016-06-06

**Authors:** Bin Du, Daniel C. Zielinski, Erol S. Kavvas, Andreas Dräger, Justin Tan, Zhen Zhang, Kayla E. Ruggiero, Garri A. Arzumanyan, Bernhard O. Palsson

**Affiliations:** Department of Bioengineering, University of California San Diego, La Jolla, CA 92093 USA; Center for Bioinformatics Tuebingen (ZBIT), Sand 1, University of Tuebingen, Tübingen, 72076 Germany; Department of Pediatrics, University of California San Diego, La Jolla, CA 92093 USA; Novo Nordisk Foundation Center for Biosustainability, Technical University of Denmark, 2800 Lyngby, Denmark

**Keywords:** Metabolic modeling, Kinetic modeling, Approximate rate laws, Michaelis-Menten kinetics, Mass action kinetics

## Abstract

**Background:**

The mechanistic description of enzyme kinetics in a dynamic model of metabolism requires specifying the numerical values of a large number of kinetic parameters. The parameterization challenge is often addressed through the use of simplifying approximations to form reaction rate laws with reduced numbers of parameters. Whether such simplified models can reproduce dynamic characteristics of the full system is an important question.

**Results:**

In this work, we compared the local transient response properties of dynamic models constructed using rate laws with varying levels of approximation. These approximate rate laws were: 1) a Michaelis-Menten rate law with measured enzyme parameters, 2) a Michaelis-Menten rate law with approximated parameters, using the convenience kinetics convention, 3) a thermodynamic rate law resulting from a metabolite saturation assumption, and 4) a pure chemical reaction mass action rate law that removes the role of the enzyme from the reaction kinetics. We utilized in vivo data for the human red blood cell to compare the effect of rate law choices against the backdrop of physiological flux and concentration differences. We found that the Michaelis-Menten rate law with measured enzyme parameters yields an excellent approximation of the full system dynamics, while other assumptions cause greater discrepancies in system dynamic behavior. However, iteratively replacing mechanistic rate laws with approximations resulted in a model that retains a high correlation with the true model behavior. Investigating this consistency, we determined that the order of magnitude differences among fluxes and concentrations in the network were greatly influential on the network dynamics. We further identified reaction features such as thermodynamic reversibility, high substrate concentration, and lack of allosteric regulation, which make certain reactions more suitable for rate law approximations.

**Conclusions:**

Overall, our work generally supports the use of approximate rate laws when building large scale kinetic models, due to the key role that physiologically meaningful flux and concentration ranges play in determining network dynamics. However, we also showed that detailed mechanistic models show a clear benefit in prediction accuracy when data is available. The work here should help to provide guidance to future kinetic modeling efforts on the choice of rate law and parameterization approaches.

**Electronic supplementary material:**

The online version of this article (doi:10.1186/s12918-016-0283-2) contains supplementary material, which is available to authorized users.

## Background

Kinetic models of biochemical networks continue to grow in scope and scale [1–7]. The promise of these models is to serve as *in silico* platforms for prediction of complex system behavior and corroboration of experimental results. Specifically within metabolism, kinetic models have the potential to elucidate the control mechanisms underlying metabolic homeostasis and regulatory responses [8–10], as well as to identify ‘flux bottlenecks’ impeding optimal performance of production strains [11]. To date, these models have been used to study such problems as the systemic effect of enzyme mutations [12, 13], metabolic bistability [10], and the coupling of signaling between metabolism and transcriptional regulation [3].

The primary challenge in kinetic modeling of metabolism is dealing with the frequent cases where data to construct detailed kinetic models is lacking [14]. This challenge is commonly addressed in part by selecting kinetic rate laws with particular approximations that reduce the number of parameters to be specified [15, 16]. If the assumptions made are valid across the conditions of interest, a consistent and predictive system should be obtainable by fitting parameters to available data [17]. Established examples of kinetic assumptions applied to enzyme reactions [5] include the quasi-steady state assumption utilized in Michaelis-Menten-type rate laws [4, 6, 18] and the lin-log approximation [2, 19] rooted in thermodynamic intuition. The degree to which these types of approximated systems represent the true system is a primary concern when choosing a modeling approach.

Here, we construct a set of kinetic models of red blood cell (RBC) metabolism using various approximate rate laws, such that their parameters are equivalent to those of the fully-described enzyme mechanistic model. We choose the red blood cell due to the large amount of available data, enabling us to use physiological enzyme kinetic parameters, reaction fluxes, metabolite concentrations, and reaction equilibrium constants. Thus, we can examine the practical importance of rate law approximations against the backdrop of a realistic system.

We utilize these models to study the effect of simplifying assumptions to the rate laws on system dynamics through simulating the network response to small transient perturbations. We additionally discuss theoretical differences in the kinetic behavior of these rate laws. Finally, we iteratively replace approximate rate laws with mechanistic enzyme kinetics to examine whether we can anticipate general dynamic effects of certain types of approximations. We purposefully chose a simple perturbation approach with mathematical response properties as output metrics, as opposed to physiological prediction accuracy, in order to simplify the task of understanding any observed correlations or lack of correlations.

## Results

### Assumptions underlying rate law approximations

**Fig. 1:**
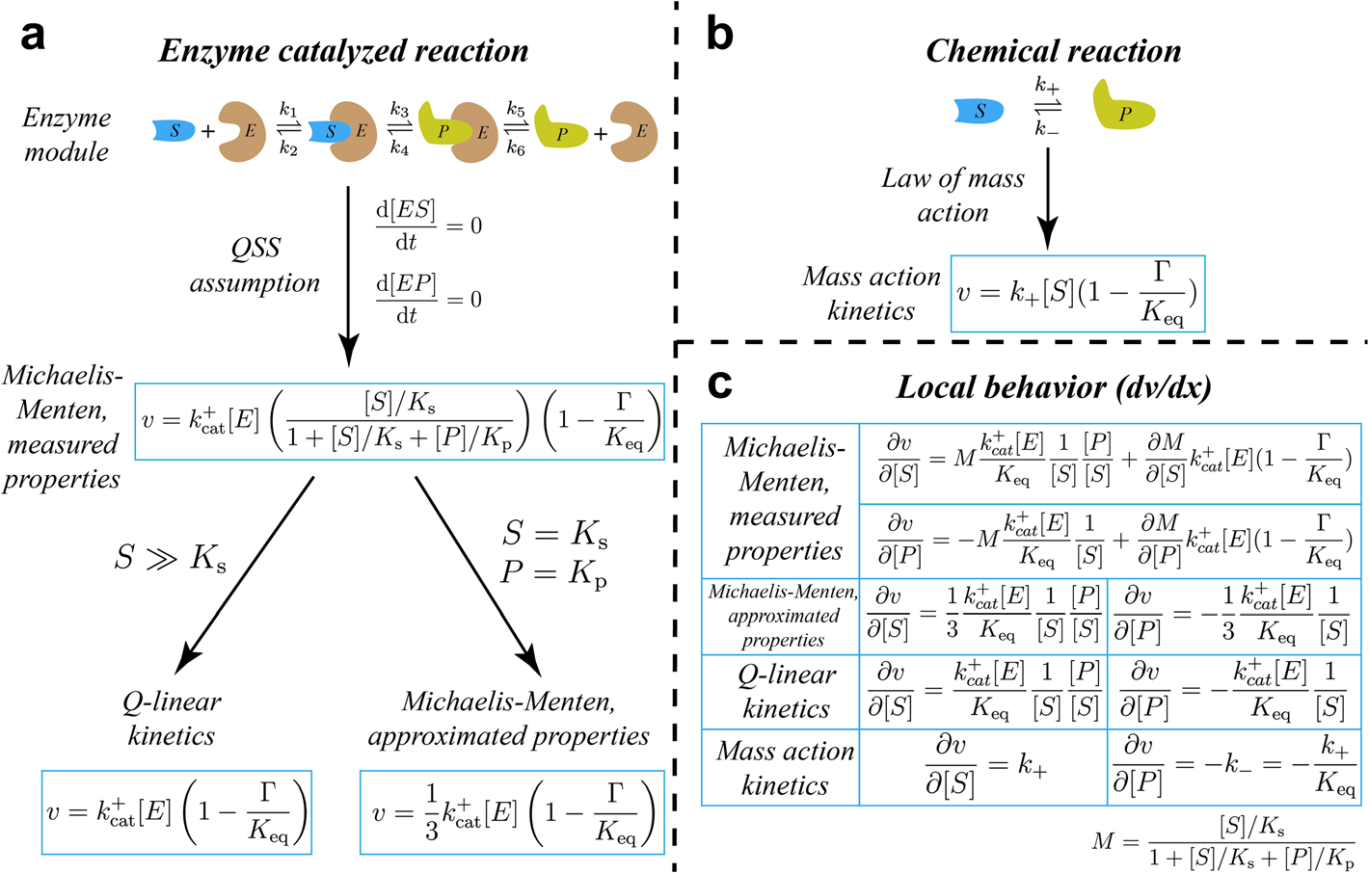
Comparison of rate laws and their resulting first derivatives. **a** Formulation of Michaelis-Menten kinetics with measured properties, Q-linear kinetics and Michaelis-Menten kinetics with approximated properties from the enzyme module with different layers of assumptions [42]. **b** Formulation of mass action kinetics based on the law of mass action for a pure chemical reaction. **c** First derivatives (reaction sensitivities) calculated from the four approximate rate laws. *K*_s_ and *K*_p_ are the Michaelis-Menten constants for the substrate and product. Γ is denoted as the mass-action ratio, which is the ratio of product concentrations over reactant concentrations in a steady state raised to the exponent of their stoichiometric coefficients. *K*_eq_ is the equilibrium constant of the reaction. *k*_*cat*_^*+*^ is the enzyme turnover rate constant. *k*_*+*_, as defined in MASS models, is the pseudo-elementary rate constant in the forward direction

In preparation for investigating rate law effects through model simulation, we first discuss the assumptions underlying the different approximate rate laws. Perhaps the most well-known kinetic assumption is the QSS assumption, normally associated with Michaelis-Menten kinetics but originated by Briggs and Haldane [20]. This assumption states that all intermediate enzyme forms do not change concentrations over time (Fig. 1a middle). Michaelis-Menten kinetics normally require Michaelis-Menten constants (K_m_s) and catalytic constants (k_cat_s) to parameterize the system, as well as metabolomics data, K_eq_s of biochemical reactions, and enzyme concentrations. The conditions for validity of the assumptions underlying this rate law have been examined in great detail [21–27].

If sufficient kinetic data is lacking, but reproducing enzyme saturation behavior is desired, an additional assumption can be made to approximate the K_m_ values. Previously it has been shown experimentally that enzyme K_m_ values tend to be similar to the in vivo concentrations of corresponding metabolites [28]. To determine whether this trend can be exploited to fill in unknown parameters, we examined the dynamic effect of using a K_m_ = x assumption to parameterize rate laws. If we additionally lack of knowledge about the enzyme reaction mechanism as well, the form of the QSS rate law equation into which parameters will be inserted is unclear. To deal with this, we can add a further assumption that the reaction follows a rapid equilibrium random order mechanism [29], following the previously suggested “convenience kinetics” formalism. We term this rate law with assumed rather than measured enzyme parameters as a Michaelis-Menten rate law with approximated properties (Fig. 1a bottom right).

Another way to address cases where enzyme-specific data is lacking is to combine the QSS assumption with a different assumption that substrates are saturated relative to their binding constants, while products and inhibitors are of negligible concentration (i.e., K_m_ < < x for substrates and activators while K_m_ > > x for products and inhibitors). This assumption effectively removes enzyme-specific parameters from the rate law and leads to a thermodynamics-driven rate law similar to what has been termed Q-linear kinetics (Fig. 1a bottom left) [30]. However, we note that Q-linear kinetics treats the mass action ratio Q as a thermodynamic variable while we treat the involved metabolites as separate variables. This Q-linear kinetics-like rate law is fully specified using only metabolomics, fluxomics, and K_eq_ data.

Finally, another method to remove the need for enzyme-specific parameters is to simply ignore the role of the enzyme and assume that the reaction behaves by simple mass action principles, and the resulting rate law is conventionally termed mass action kinetics [5, 31]. This form effectively assumes that the reaction behaves as a pure chemical reaction with a single transition state (Fig. 1b). As with Q-linear kinetics, mass action kinetics requires relatively few parameters to describe the system, namely metabolomics, fluxomics, and K_eq_ data.

The benefit of requiring fewer parameters is the major motivation for applying these simplified rate laws; however, before using them, we carefully examine whether they are able to accurately capture the dynamics of a model constructed of detailed enzyme modules. We might expect two general cases where rate law approximations should be successful. First, in cases where the underlying assumptions are valid, the rate law approximations should show accurate behavior provided that the assumptions are not violated substantially throughout the simulation. Second, if the rate laws are not the most important factor determining the dynamic behavior of the network, we would expect the use of an approximation to have little negative effect. For example, some of the rate laws may behave similarly near to equilibrium. In the course of this investigation, we will seek to identify both the degree to which approximate rate laws can reproduce the behavior of the true model, as well as the causes of this agreement or lack thereof.

### Differences in mathematical behavior between rate laws

To place the subsequent results of simulating the various kinetic models in theoretical context, we briefly discuss differences between the analytical structures of the various rate laws. We focus on two key points: 1) the ability of the rate law to exhibit the ‘saturation’ behavior that is characteristic of enzyme kinetics, and 2) the properties of the first derivative of the rate law, which defines the local dynamic behavior of the system.

Each rate law exhibits different behavior as metabolite concentrations approach infinity. For example, the Michaelis-Menten kinetics with measured properties exhibit the well-known saturation behavior due to the hyperbolic form, such that v = v_max_ as x approaches infinity. A mass action enzyme module exhibits the same behavior due to the constant total enzyme, placing a constraining relationship between the fluxes of individual reaction steps. The manner in which saturation is achieved between a full mass action enzyme module and the Michaelis-Menten kinetics is thus mathematically different.

In contrast to Michaelis-Menten kinetics with measured properties and enzyme module of mass action rate laws, the non-module mass action and Q-linear rate laws do not exhibit saturation behavior. Mass action kinetics will approach positive or negative infinity as substrate or product concentrations, respectively, approach infinity. Meanwhile, Q-linear kinetics exhibit asymmetrical saturation properties. The flux v will correctly have a maximum of v_max_ if the substrate concentration is maximized, but will incorrectly have a minimum of negative infinity if the product concentration is maximized. This asymmetry is known and proponents of the rate law suggest that the rate law only be used in a range near equilibrium [19], which is not possible to guarantee in real perturbations. For this reason, it is expected that the Q-linear kinetics and mass action kinetics will exhibit large deviations from the true mass action module system when perturbation of the saturation state of the enzyme is an important feature of the dynamic response.

Examining the first derivatives of the reactions is a straightforward analytical approach to anticipating dynamic differences between the rate laws (Fig. 1c). From the analytical form of the rate law first derivatives, it is clear that the local dynamics between each type of rate law will be potentially substantially different, with numerical values dominated by different parameters in each case. The expressions for gradients obtained from the Michaelis-Menten kinetics with measured properties are complicated and multiple parameters play a role in affecting the numerical gradient values. The Michaelis-Menten kinetics with approximated properties and Q-linear kinetics rate laws have almost the same composition of their first derivatives, determined by enzyme turnover rate constant, the equilibrium constant and substrate and product concentrations. On the other hand, the local dynamic gradient in mass action kinetics is determined by the pseudo-elementary rate constants and equilibrium constant.

### Construction and general properties of mass action modules for ten enzymes

**Fig. 2:**
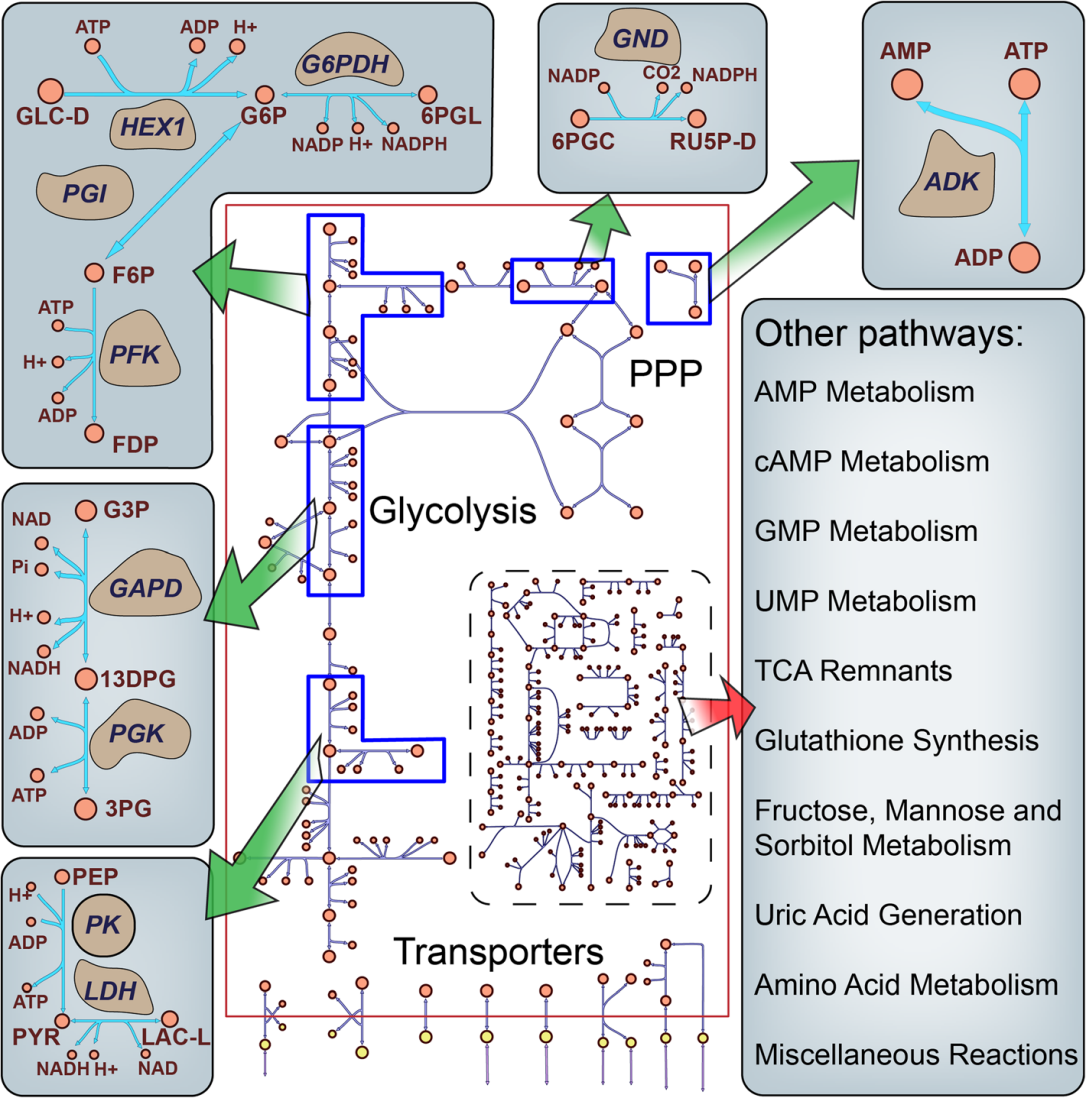
Schematic of the enzyme modules incorporated into the RBC metabolic network [33]. The ten modules constructed were primarily located in glycolysis and the pentose phosphate pathway. Other pathways were included as Q-linear kinetics approximations

We first constructed enzyme ‘modules,’ consisting of full mass action descriptions of enzymatic reaction mechanisms, for ten key enzymes in RBC central metabolism utilizing measured data for these enzymes (Fig. 2, Table 1). An enzyme module consists of mass action rate laws for all known reaction steps such as substrate binding, catalytic conversion, and product release, as well as any activator or inhibitor binding (Fig. 1a top). An enzyme module describes the detailed mechanism of enzyme catalysis and characterizes the dynamics of the enzymatic reaction subject only to certain basic assumptions such as deterministic behavior and a well-mixed solution [32]. The enzyme module requires a large number of parameters, including metabolomics data, equilibrium constants (K_eq_s), enzyme concentrations, and rate constants of individual enzymatic reaction steps, to fully describe the dynamics of the system. We used these ten enzyme modules as a ‘gold standard’ for later comparison with approximate rate laws.

**Table 1:** General description of the constructed enzyme modules

Enzyme name	Module size (metabolites × reactions)	Regulators (mechanism of action)
Phosphogluconate dehydrogenase (*GND*)	13 × 9	NADPH (PI)
Lactate dehydrogenase (*LDH*)	10 × 6	N/A
Glucose-6-phosphate dehydrogenase (*G6PDH*)	12 × 7	ATP (CI), NADPH (PI)
Glyceraldehyde 3-phosphate dehydrogenase (*GAPDH*)	27 × 27	3PG (AI), G3P (AI)
Hexokinase (*HEX1*)	10 × 6	23DPG (CI)
Pyruvate kinase (*PK*)	30 × 34	FDP (AA), ATP (PI, AI)
Phosphofructokinase (*PFK*)	40 × 44	ADP (PI), ATP (AI), AMP (AA)
Phosphoglycerate kinase (*PGK*)	13 × 9	ATP (PI), 3PG (PI), 23DPG (CI)
Adenylate kinase (*ADK*)	8 × 5	N/A
Glucose-6-phosphate isomerase (*PGI*)	5 × 3	N/A

*PI* product inhibitor, *AI* allosteric inhibitor, *AA* allosteric activator, *CI* competitive inhibitor

### Construction of approximate rate laws

In this study, we examined four approximate rate laws to compare to the fully-described enzyme modules. Those four rate laws are: 1) Michaelis-Menten kinetics based on the quasi-steady state (QSS) assumption for the true enzyme module with measured enzyme parameters, 2) an assumed rapid-equilibrium random-order Michaelis-Menten rate law ignoring regulation and with K_m_ values being approximated as equal to the concentrations of corresponding metabolites, to simulate the effect of unknown data, mechanisms, and regulation, 3) a rate law previously, termed Q-linear kinetics [30], containing only thermodynamic effects that results from a further metabolite saturation assumption, and 4) a rate law based on the mechanism of chemical mass action that effectively ignores the role of the enzyme in the reaction [5].

### Construction of an approximate rate law scaffold model

We first constructed a cell-scale model of RBC metabolism using approximate Q-linear rate laws to serve as a scaffold model for analysis. Our approach was to insert the ten constructed enzyme modules into this scaffold, and compare this model behavior to that of models generated with different approximate rate laws substituted into those same ten reactions. The model was constructed using steady-state metabolite levels from plasma and intracellular erythrocyte metabolomics data from a fasting state [33]. The model contains 169 metabolites and 143 reactions, covering glycolysis, the pentose phosphate pathway, amino acid metabolism, and other pathways (Additional file 1). Detailed information of the kinetic model can be found in Additional file 1: Figure S1.

### Designing a simulation-based kinetic analysis workflow

A straightforward way to estimate the similarity of behavior between different rate laws is to simulate the response of each model to perturbation. A perturbation in this case denotes the change of certain metabolite concentrations at time t = 0, after which the system is allowed to simulate through a long enough time such that the original steady state is once again reached. For example, we perturbed the concentrations of ATP, ADP and P_i_ at the same time to simulate the hydrolysis of ATP in the system.

**Fig. 3:**
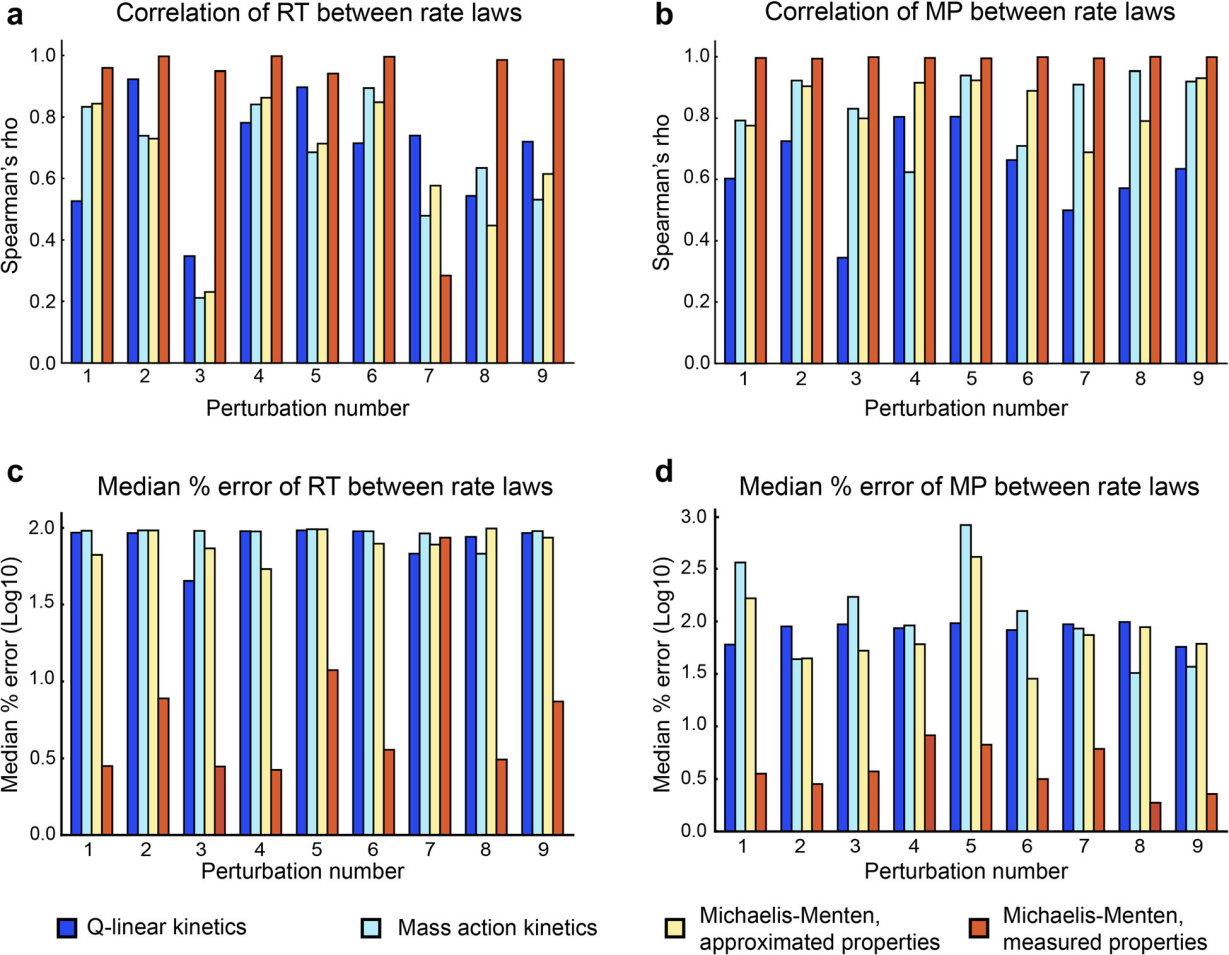
Simulation comparison of four simplified rate laws against a reference module containing detailed enzyme mechanism kinetics (enzyme modules). The responses of metabolites under different perturbations were compared between four simplified rate laws and the enzyme module. **a** Correlation of metabolite relaxation time. **b** Correlation of metabolite maximum perturbation. **c** Median percent errors of metabolite relaxation time. **d** Median percent errors of metabolite maximum perturbation. Nine different perturbations labeled from 1 to 9 were performed. 1, ATP, ADP and P_i_ perturbation; 2, NAD and NADH perturbation; 3, 23DPG perturbation; 4, 3PG perturbation; 5, PYR perturbation; 6, FDP perturbation; 7, PRPP perturbation; 8, MAN6P perturbation; 9, R5P perturbation. Spearman’s rho: Spearman’s rank correlation coefficient. The simulations were performed on the whole-cell kinetic model of erythrocyte constructed by Bordbar et al [33]

Two key decisions in such an analysis are the choice of perturbation and the choice of output variable to observe. In this study, we perturbed both metabolites directly involved in as well as distant from the constructed enzyme modules. The list of perturbations can be found in Fig. 3. To define output variables of interest, we created two metrics, the maximum perturbation (MP) and the relaxation time (RT). The MP is largest percent change in concentration compared to the steady state concentration that occurred during the simulation. Then, to calculate the RT of a metabolite, we identify the last time point at which the deviation from the steady state concentration is at least 5 % of the MP.

One final decision in the simulation workflow is the size of the perturbation to use. As mentioned previously, the rate laws chosen differ in both saturation properties, which are non-linear features of the rate laws, and local dynamic properties, which are linear features of the rate law. It appeared to be a trivial result that saturating and non-saturating rate laws will exhibit very different behavior for large deviations where non-linear effects play a significant role. However, understanding the origin and nuances of such deviations is complex, and we sought to achieve a simpler goal as a baseline investigation. To avoid such obvious effects dominating our findings, we intentionally chose small perturbations to minimize saturation effects and instead focus on determining the importance of the linear/local differences between rate laws.

### Numerical comparison of rate laws

The final workflow was to perform nine different small perturbations on the system with different rate laws and characterized the response of metabolites in terms of RT and MP (Fig. 3a–b). Calculating the Spearman correlation for MP and RT of module metabolites between rate laws, we found that the Michaelis-Menten kinetics with measured properties behaved substantially better on both metrics compared to other rate laws. Median percent errors for MP and RT of module metabolites confirmed this trend (Fig. 3c–d). Additionally, we found that the Michaelis-Menten rate law with approximated properties performed no better than the Q-linear kinetics and mass action kinetics. This indicates that the K_m_ = x assumption (x being the concentration of the corresponding ligand) is not sufficiently correct to capture the dynamics of the original enzyme module. Notably we did not include known regulation of these enzymes in this approximate rate law, and further investigation of the behavior of models with the addition of these regulatory events with an analogous K_d_ = x assumption may be warranted. We note that these conclusions regarding the suitability of approximate rate laws are not due to the choice of model underlying the analyses.

We repeated these analyses on a previously published model of the red blood cell, smaller scale but composed entirely of mechanistic enzyme mechanisms [34]. We iteratively substituted in different approximate rate laws and verified the identified trends, where Michaelis-Menten with measured properties performs substantially better than the other approximations but all approximations retain positive correlation to the true model (Additional file 1: Figure S2). We also verified the results using larger perturbations, suggesting that non-linearity of the perturbation response does not strongly affect the trends (Additional file 1: Figures S3-4). However, as an exception to the general trends, we did identify rare perturbations where Michaelis-Menten rate laws with measured enzyme properties performed noticeably worse than more approximated rate laws (Additional file 1: Figures S3-4). We attribute these cases to slow internal dynamics within the enzyme module, causing the quasi-steady state assumption to become invalid. However, these effects were difficult to isolate and we did not investigate these cases further due to their infrequency.

One key control in the study is to determine whether uncertainty in parameters significantly impacts the conclusions of analyses. To address this, we conducted Markov chain Monte Carlo (MCMC) convex sampling of steady-state fluxes given physiological ranges on metabolite uptakes and secretions [33]. Similarly, we conducted MCMC sampling of metabolite concentrations subject to a constraint on the feasibility of the concentrations with respect to the 2^nd^ law of thermodynamics [35]. We then combined sampled fluxes and concentrations and calculated rate constants for mass action rate laws for each reaction. The resulting rate constants are shown in Additional file 1: Figure S5A. It is seen that the variation in rate constants due to flux and concentration uncertainty is small compared to the variation between rate constants of different reactions in the majority of cases. We also performed several simulations on models with these sampled rate constants, and found little variation in the RT or MP of metabolites across sampled models (Additional file 1: Figures S6-9). Thus, it appears that experimental uncertainty in fluxes and concentrations, and the resulting uncertainty on estimated rate constants for simplified rate laws, is not a major concern in making claims about the dynamics of the network.

**Fig. 4:**
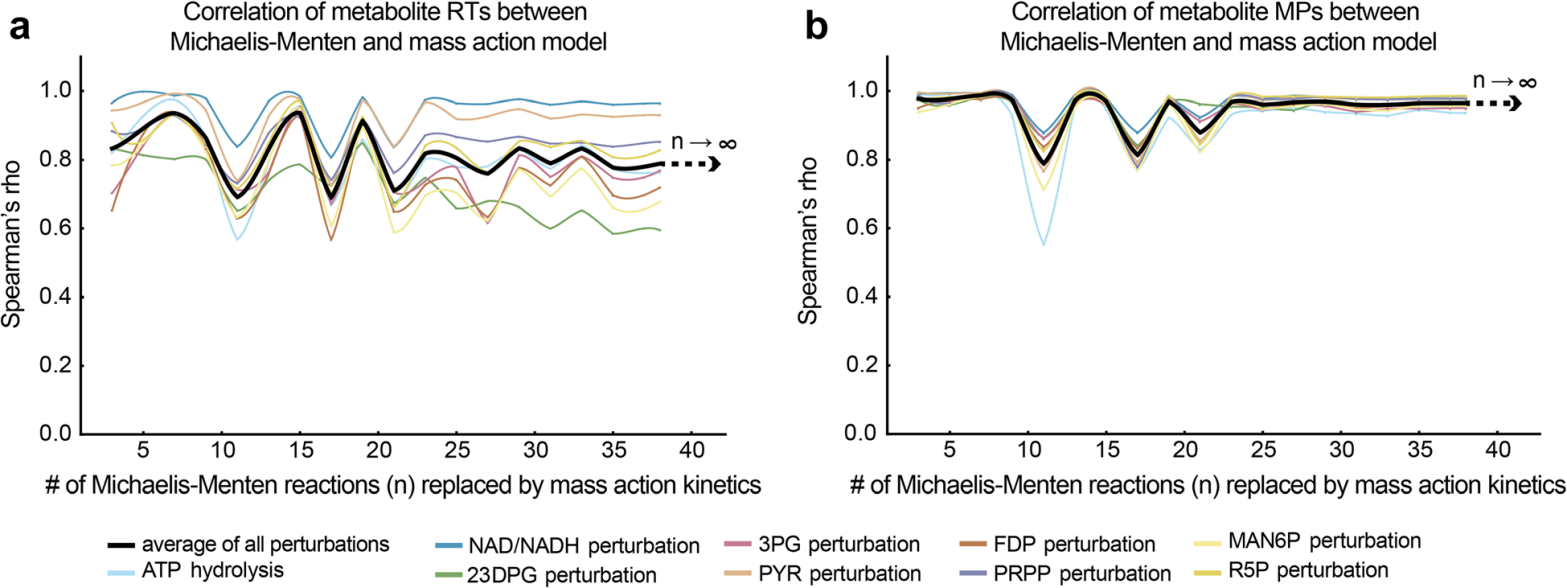
Iterative replacement of Michaelis-Menten kinetics with measured properties by mass action kinetics. An increasing number of Michaelis-Menten kinetics rate laws with measured parameters were replaced by mass action kinetics, and the RT and MP of affected metabolites were calculated. The correlation of metabolite RT and MP between Michaelis-Menten kinetics and mass action kinetics fluctuated initially but gradually stabilized as more reactions were replaced with mass action kinetics. The black line is the average correlation of all nine perturbations performed. **a** Correlation of metabolite RTs between Michaelis-Menten and mass action model. **b** Correlation of metabolite MPs between Michaelis-Menten and mass action model. Spearman’s rho: Spearman’s rank correlation coefficient. The simulations were performed on the whole-cell kinetic model of erythrocyte constructed by Bordbar et al [33]

Since the simplified rate laws introduces noticeable discrepancies in dynamic behavior, we wanted to determine whether these discrepancies would continue to increase as simplified rate laws are applied to more reactions until the correlation completely disappears, or whether the approximate model behavior would stabilize at some positive correlation to the true model. Based on the previous observation that Michaelis-Menten kinetics with measured properties closely resembled the true model, we set up a simple test case with as many reactions specified with Michaelis-Menten kinetics as possible (38 out of 168 reactions [33]) and then iteratively replaced them with mass action kinetics. We compared the RT and MP of the substrates and products of these reactions when a random set of reactions had their rate laws changed from Michaelis-Menten to mass action kinetics. We found that the correlation of RT and MP of metabolites between Michaelis-Menten and mass action kinetics stabilized as more reactions had their rate laws substituted (Fig. 4). Since the discrepancy ceases to grow after a certain point, it appears likely that models with constructed entirely of simplified rate laws still be useful approximations of the real system, at least for small perturbations.

### Effects of flux and concentration steady-state on network dynamics

**Fig. 5:**
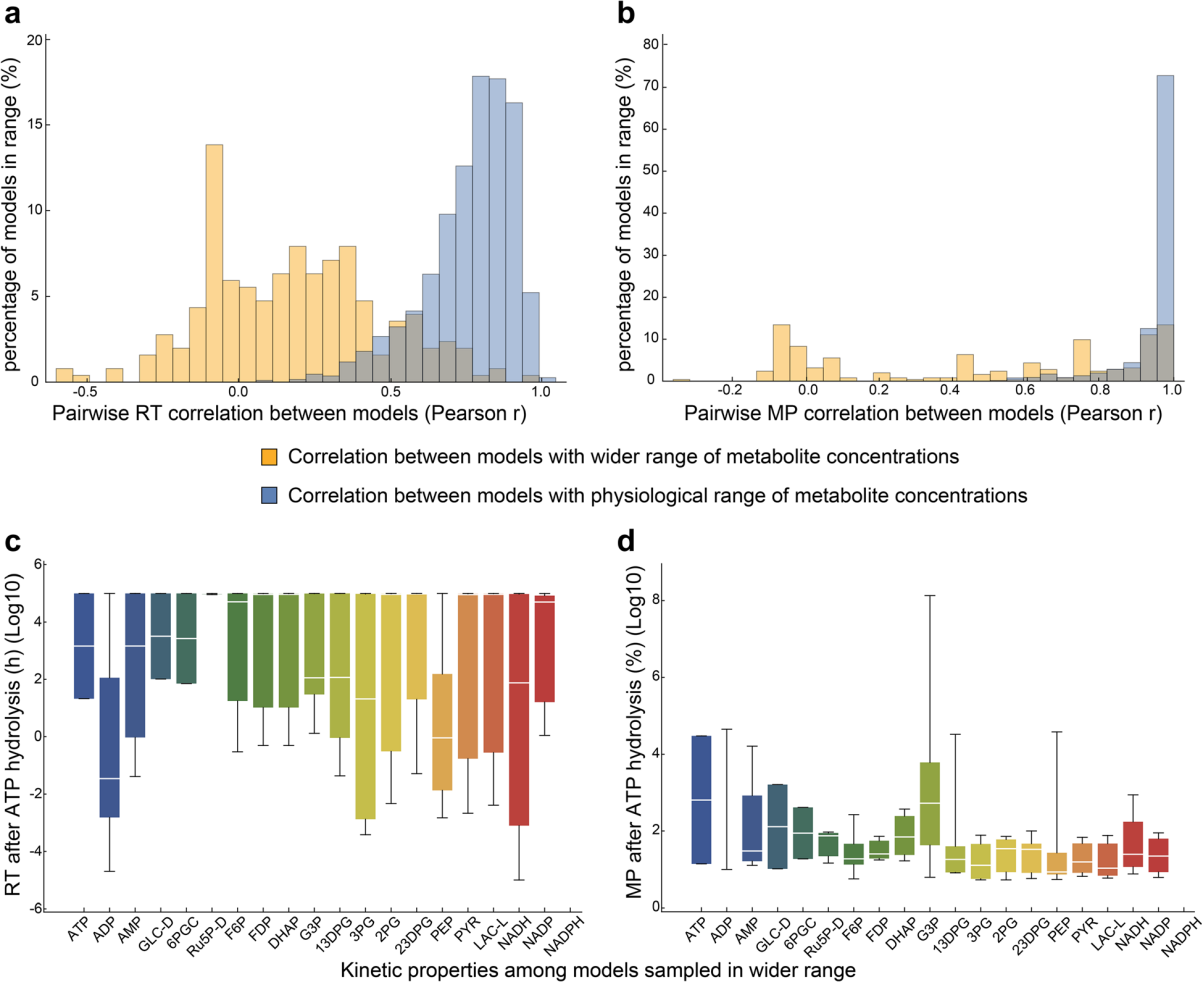
Kinetic properties of models sampled with models sampled with physiological concentrations and fluxes compared to models sampled in wider ranges of concentrations and fluxes. First, 63 models were built with metabolite concentrations and fluxes sampled from physiologically relevant range. Then, 23 models were constructed with a wider range of metabolite concentrations (10^−8^ to 10^5^ mM) and fluxes. ATP hydrolysis was chosen as a reference perturbation as the perturbation on all models and RT and MP of the metabolites was calculated. **a** Distribution of pair-wise Pearson correlation coefficients of metabolite RTs for models sampled with wider concentration and flux ranges and models sampled with physiologically relevant ranges. **b** Distribution of pair-wise Pearson correlation coefficients of metabolite MPs for models sampled with wider concentration and flux ranges and models sampled with physiologically relevant ranges. **c** Distribution of metabolite RTs for models sampled with wider concentration and flux ranges. **d** Distribution of metabolite MPs for models sampled with wider concentration and flux ranges. The sampling and simulations were performed on the whole-cell kinetic model of erythrocyte constructed by Bordbar et al [33]

We then investigated the source of the positive correlation between fully approximate models and the true model. As both models share the same initial steady state, in terms of reaction fluxes, metabolite concentrations, and reaction equilibrium constants, we sought to determine whether these values were essential to the dynamic consistency we observed across rate laws. The flux and concentration state of the cell play a role in determining the dynamic structure of the network. For example, large metabolite pools will be changed slowly by small fluxes, and vice versa, giving some expectation of fast and slow dynamics within the network. We wanted to investigate the degree to which network dynamics are determined by the initial flux and concentration state, as opposed to the choice of rate law. To this end, we sampled reaction fluxes and metabolite concentration within physiological ranges, and then in wider ranges. In contrast to changing rate laws, we found that widening the sampling range on fluxes and concentrations greatly impacted the dynamic response of metabolite throughout the network. For example, metabolite MP and RT subject to ATP hydrolysis perturbation showed weaker correlations within models sampled with wider concentration and flux ranges compared to those from models sampled with physiological concentration and flux ranges (Fig. 5a–b). We also found that the distribution of metabolite RT and MP under ATP hydrolysis perturbation spanned a much larger range for models sampled with wider concentration and flux ranges (Fig. 5c–d). Thus, it appears that the origin of the dynamic consistency across rate laws does indeed lie within the order of magnitude differences across reaction fluxes and metabolite concentrations throughout the network.

### Dependence of the effect of rate laws approximations on reaction properties

**Fig. 6:**
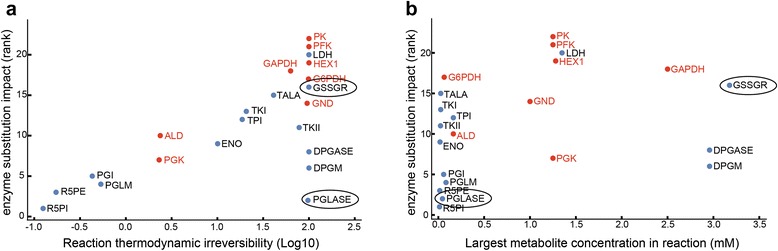
Reaction properties affecting the impact of reaction rate law approximations. **a** Enzyme substitution impact (rank) against reaction thermodynamic irreversibility (Log10). Reaction thermodynamic irreversibility is calculated as (reaction equilibrium constant - mass action ratio)/reaction equilibrium constant. Lower rank score meant less change in dynamic response when the module is replaced by mass action kinetics. Reactions highlighted in red indicate presence of regulation. Circled reactions are outliers of the general trends. *PGLASE* is irreversible but shows low impact upon reaction rate law approximation. *GSSGR* has a large substrate concentration, yet still shows significant impact upon reaction rate law approximation. **b** Enzyme substitution impact (rank) against largest metabolite concentration in the reaction. Red and circled reactions are the same as in panel (**a**). The simulations were performed on the model constructed based on Mulquiney et al [34]

We have showed that, while models constructed with approximate rate laws still hold valuable dynamic information due to the constraining effects of physiological flux and concentration differences, there is still a substantial increase in model accuracy from inclusion of additional kinetic information such as in a Michaelis-Menten rate law with measured properties. However, the question is still open of whether certain reactions are more necessary to model accurately than others. To probe this question, we began with a fully-defined mechanistic model [34], substituted each reaction in turn with a mass action approximation, and determined the effect on network dynamics. Clear trends emerged. First, reactions farther from equilibrium showed a larger effect from rate law approximation (Fig. 6a). This is intuitive as irreversible reactions tend to be regulated allosterically, but the trend existed even for non-regulated enzymes. Second, certain reactions with metabolites that have high concentration tend to show a smaller effect by substitution of rate law approximation as well. For example, the enzymes *DPGASE* and *DPGM* are thermodynamically in an irreversible state but the high concentration of 23DPG creates a large slow moving pool that causes the dynamics of the network to be insensitive to the choice of rate law for these enzymes (Fig. 6). However, there remain some unexplained cases, where reactions have one or both of these properties but rate law approximations result in effects outside of the general trend previously observed. For example, the enzymes *PGLASE* and *GSSGR* are clear outliers. This suggests that additional properties exist, such as network context given particular perturbations of interest, that may provide additional cases where rate law approximations work well.

### Evaluating the consistency of effects of single enzyme mechanism substitutions throughout the network

One natural question to arise is whether it is possible to anticipate the changes to dynamic properties that occur when introducing enzyme mechanisms with particular features, such as allosteric regulation or a location upstream of a metabolite of interest, in place of an approximate rate law. For example, there exist some rules of thumb when dealing with small feedback networks, such as the role of negative feedback in increasing system response time, that might be applicable in these networks. However, we did not find such rules of thumbs to be reliable in the cases we examined.

In the case of the importance of network localization, for the nearby enzymes *PK* and *PGK*, there was no general trend observed in metabolite MPs and RTs under ATP hydrolysis perturbation following single module addition of *PK* or *PGK* (Additional file 1: Figure S10, Table S1). For example, the addition of the *PGK* module slightly decreased the MP of lactate compared to no module while the addition of the *PK* module caused an increase in the MP of lactate, while from a structural standpoint we might expect the lactate node to have similar responses to the introduction of either enzyme mechanism. Along the same lines, the RT of 23DPG increased when adding the *PGK* module but decreased when adding the *PK* module. In addition to looking at the effect of different enzyme substitutions for a particular perturbation, we also looked across different perturbations for the same enzyme substitution. Specifically, we characterized the response of metabolite PYR under different perturbations upon the addition of the *PK* module and did not observe any general trend in the change of response (Additional file 1: Figure S11, Table S2).

As a case study for the effect of adding allosteric regulation, we chose the *HEX1* enzyme module, which contains 23DPG as a feedback inhibitor. We performed multiple perturbations on *HEX1* module with and without regulation and characterized the change in the dynamic response of the substrates and products of the enzyme. We found that G6P showed an increase in RT following addition of the feedback inhibitor, indicating that G6P relaxes more slowly following the addition of the inhibitor. The increase was also observed in metabolites downstream of the module. Meanwhile, G6P and F6P specifically showed an increase in MP with the addition of feedback inhibition (Additional file 1: Figure S12A-B). These observations appear contrary to the effect of feedback inhibition in simple feedback loops, where RT and MP decrease due to the effect of the inhibition [36]. This contradiction might be due to other interactions within the model, where metabolic reactions are usually nonlinear due to metabolites shared across multiple reactions. We performed the same analysis on the *GAPDH* module with 3PG as a feedback inhibitor. However, in this case we found a decrease in RT on FDP and G3P when the feedback inhibition was added, as well as a decrease in MP on 3PG and PEP (Additional file 1: Figure S12C-D). The two case studies above showed that the feedback inhibition can cause quite different responses in different modules and the effect of regulatory mechanisms should be carefully considered on a case by case basis.

We also analyzed the effect of feedforward activation as an additional example of regulation. The example we studied the *PK* module with FDP as a feedforward activator. We found a decrease in RT for PYR in the *PK* module as well as a few metabolites upstream of the *PK* module, such as G6P, F6P, FDP, G3P and 3PG (Additional file 1: Figure S13). Those metabolites also had a decrease in the MP (except 3PG and PYR). Again, this is contrary to the commonly observed effect of a simple feedforward loop, where RT and MP subsequently increase following addition of a feedforward activator [36]. Similar to the feedback inhibition, such contradiction may be attributable to more complex interactions within the metabolic network.

Overall, we showed that module addition can qualitatively affect the dynamics of related metabolites, but the quantitative effect can vary from case to case, possibly due to associated reaction and network connectivity properties. Therefore, it is difficult to predict any kind of consistent change moving from an accurate mechanistic description of enzyme catalyzed reactions to more approximate rate laws in specific cases.

### Physiological and enzyme activity perturbations

Finally, while results so far were generated using perturbations of largely academic purpose, such as spontaneous internal metabolite changes, we sought to verify our results on perturbations of greater physiological meaning. First, we performed several simulations on decreased enzyme activity, in the form of a lower enzyme concentration or lowered catalytic rate constant, for the enzymes *G6PDH*, *PGK*, and *PK*, and verified the rate law trends identified thus far (see Methods). For example, the relative metabolite concentrations across different levels of *G6PDH* activity were the same between enzyme module and Michaelis-Menten rate law with measured properties, while other rate laws showed noticeable differences (Additional file 1: Figure S14). We made similar observations on relative metabolite level change across *PK* or *PGK* activity change, except that in *PGK* all rate laws behaved closely to the enzyme module (Additional file 1: Figures S15-16). Then, we mimicked a previous study on an oxygen deprivation perturbation [37], and found that Michaelis-Menten rate law with measured properties was able to match exactly the dynamics of enzyme module, outperforming other approximated rate laws. However, none of the models quantitatively matched the experimental data well, suggesting confounding parameterization or model scope issues (Additional file 1: Figure S17).

## Discussion

In this work, we constructed a kinetic model of RBC metabolism with a mechanistic description of ten enzymatic reactions and compared the dynamic properties of the mechanistic model with those of several commonly proposed simplifying assumptions. We found that the Michaelis-Menten kinetics with measured properties yields a consistently good approximation of the full system, while the Q-linear kinetics and mass action kinetics can show substantial discrepancies. Furthermore, we formulated another Michaelis-Menten-type rate law in an attempt to simplify the Michaelis-Menten kinetics given limited data available, based on a K_m_ = x_ss_ assumption with a rapid-equilibrium random order binding reaction scheme. However, this approach failed to show improved agreement in dynamics with the enzyme modules over other approximations. We attribute the positive correlation of even the most approximate rate laws with the true model as due to the important effect that reaction flux and metabolite concentration differences play in the network dynamics.

Obtaining enzyme kinetic parameters continues to be a core issue hindering the development of practical large-scale kinetic models of metabolism. Databases such as BRENDA [38] continue to aggregate studies on the kinetic properties of enzymes for various organisms. However, not only are the collections of the most common kinetic parameters (K_m_s and k_cat_s) often incomplete and measured under non-physiological conditions, but there is a separate issue with the additional parameters that are required to parameterize a mass action mechanistic description of a reaction (which we term an enzyme module). Full specification of kinetic parameters is experimentally intensive but theoretically possible, and some enzymes such as *PFK* have been characterized in great detail in particular organisms, including pH and temperature dependence of parameters. However, the difficulty in determining these parameters and uncertain immediate value of the data, evidenced by lack of practical applications of resulting kinetic models, is likely the main reason these data are not routinely being generated. In this study, we show both the value of fully-defined enzyme mechanism as well as rate law approximations, and thus it appears that the appropriate rate law to use should continue to be determined by the goals of the modeler.

On the note of the design of this study, we note that kinetic models can be analyzed from numerous angles. Much work thus far has focus on the dynamic control of metabolic states. This goal is of great importance, but due to the non-linear and complex nature of such control, we targeted our investigation on a simpler task of understanding transient responses to small perturbations in the metabolic network. Experimentally measuring such transients, i.e., dynamics of metabolite concentrations, is challenging and fundamentally limited by sampling frequency and metabolism quenching time. However, we chose to focus on these perturbations as they are the most simple to understand mathematically. Further studies looking at the effect of rate law approximations on more intricate dynamic properties, such as the non-linear control of steady-state changes following enzyme inactivation, are extremely desirable if they can be conducted in a rigorous way.

In our comparison of rate laws, we showed that the Michaelis-Menten kinetics with measured properties gives a good approximate of the full system when comparing the relaxation time and maximum perturbation of the metabolites. Thus, discrepancies due to ignoring dynamics of individual enzyme forms do not appear to be a significant issue. This success in approximation is likely due to the combination of the small concentrations of most enzyme forms relative to metabolite concentrations, a requirement for the validity of the QSS assumption [21], as well as the relatively large rate constants for reactions involved in enzyme regulation (effector binding) and structural transitions. For enzymes with larger concentrations and slow regulatory enzyme motions, there would likely be substantial discrepancies from using a QSS assumption. We also found that additional approximations from assuming saturation or neglecting enzyme behavior entirely cause substantial dynamic and structural issues. While these methods are attractive due to obviating the need for enzyme-specific parameters, the potential drawbacks may preclude their use. As an alternative, assumptions about enzyme parameters can be made in place of assumptions about rate laws. For example, one study has shown that metabolite concentrations tend to hover around the K_m_s for corresponding enzymes [28], which could be a useful assumption for modeling in lieu of sufficient data. However, in practice, we found this assumption to be insufficient to recapitulate enzyme kinetic behavior, as deviations of the real data from this assumption were sufficiently large to induce substantial differences in behavior.

We showed that adding a module can bring qualitative effects to the dynamics of related metabolites. However, the quantitative effects have to be examined in a context specific manner, possibly due to the associated reaction property or network connectivity. We also showed that the addition of regulations, such as feedback inhibition and feedforward activation, can cause dynamic behavioral changes different from those of simple genetic circuits. Taken together, we would advise a detailed mechanistic description for enzyme catalyzed reaction is likely a necessity for predicting system dynamics with reasonable accuracy.

There are two additional possible issues associated with modeling enzyme kinetics using an enzymatic mass action approach. The first is the estimation of kinetic parameters within the module. The current available experimental data on the enzyme include K_m_s, v_max_ and K_d_s. However, those data are not sufficient to solve for the rate constants of specific enzymatic steps in the module. Thus, a good fitting approach is necessary to obtain a set of rate constants that accurately recapitulate the existing experimental data. The second problem is associated with the simulation of the system containing multiple modules. A possible stiffness issue can occur when integrating the ODE equations during dynamic simulations. This might be due to the large difference in orders of magnitude between metabolite concentrations and enzyme intermediate concentrations. In this case, we would advise normalizing the enzyme concentrations to the same level as metabolite concentrations and adjust the corresponding rate constants. However, one needs to be careful with the magnitude of change in enzyme concentrations as we found that different changes can cause different dynamic responses. Looking forward, addressing these issues will be essential to make progress toward bottom-up construction of kinetic models of metabolism.

## Conclusions

The work here explored the validity of using approximate rate laws with varying levels of assumptions in the context of a cell-scale RBC kinetic model. We found that the Michaelis-Menten rate law based on quasi-steady state assumption was able to recapitulate the dynamic behaviors of the mechanistic model consistently as long as measured parameters were used. Rate laws that are derived from further approximations on Michaelis-Menten kinetics or ignore the role of the enzyme showed substantial discrepancies in dynamic behaviors compared to the mechanistic model. However, we found that the errors associated in these approximate models appeared to stabilize as more reactions were replaced by approximate rate laws, suggesting that even fully approximate models can contain useful information. This appears to be due to the dominant effect that the order of magnitude differences in reaction fluxes and metabolite concentrations have on the dynamic structure of the network. Still, we also found that replacing approximate models with the detailed mechanistic enzyme module can bring unpredictable quantitative effects to the system, suggesting a clear benefit of constructing mechanistically detailed enzyme modules when possible. The work here should aid the choice of rate laws and parameterization approaches in future kinetic modeling efforts.

## Methods

All work was done in Mathematica. We used the MASS Toolbox kinetic modeling package (https://github.com/opencobra/MASS-Toolbox) for model construction and simulation. The RBC metabolic network with enzyme modules incorporated is available in Mathematica file format.

### Construction of enzyme modules

The mass action rate law was used for reactions in enzyme modules, and the formulation can be found in Jamshidi et. al. [5].

The steps for constructing enzyme modules are as follows:

1. Define elementary reactions and obtain their equilibrium constants from literature
2. Formulate the steady state mass balances for enzyme forms and solve them symbolically in terms of parameters of the reactions
3. Substitute the symbolic enzyme forms into the equation of total enzyme concentration and approximate the rate constants of the reactions given a particular flux state
4. Calculate concentrations of individual enzyme forms given the estimated rate constants

For enzyme module with regulation, an additional enzymatic step was added in which the effector molecule (activator or inhibitor) is bound to a particular enzyme form.

The data used for module construction can be found in Additional file 1: Table S3.

### Simulation of the network with the incorporated enzyme modules

The constructed modules were added into the RBC metabolic network [33] for further analysis. For incorporation of a specific module (e.g., *PFK* module), all the reactions in the module were added into the metabolic network and the original metabolic reaction (*PFK* reaction) was removed.

Before dynamic simulations, the steady state metabolite concentrations were set as the initial conditions of the system. For a particular perturbation, a change on certain metabolite concentrations were applied at time 0 and the subsequent simulation was conducted through numerical integration of the ODE equations. The system was allowed to simulate to over 100,000 h to regain the steady state concentrations.

### Calculation of maximum perturbation and relaxation time

Given a concentration profile from simulation, the maximum perturbation is the largest percent change in concentration compared to the steady state concentration for a particular metabolite. The relaxation time is defined as the last time point at which the deviation from the steady state concentration is 5 % of the maximum perturbation. Specifically, when calculating the relaxation time, we traced backwards by starting from the concentration at a ‘long enough’ time (e.g., 100,000 h) and calculated the difference between the concentration at a particular time and the steady state concentration until the relaxation time was identified.

### Constructing a model full of enzyme modules

We used the scope (Mulquiney et al [34] Scheme 1) and kinetic data (Mulquiney et al [34] Appendix) to construct a model full of enzyme modules. Specifically, the model contains 22 modules, mainly falling in glycolysis and pentose phosphate pathway. The enzyme modules were constructed based on the method previously described. We also added in the enzyme module for hemoglobin, which can be loaded from MASS Toolbox kinetic modeling package (https://github.com/opencobra/MASS-Toolbox). There are extra 13 reactions in the model that we did not build enzyme modules for. They are export/import reactions, generic metabolic reaction without specific reference to an enzyme and reactions with zero flux. Specifically, they are AMP export reaction, AMP import reaction, CO2 export reaction, glucose import reaction, proton export reaction, water export reaction, lactate export reaction, O2 export reaction, pyruvate export reaction, ATP hydrolysis reaction, glutathione redox reaction, NADH redox reaction, adenylate kinase reaction.

### Enzyme activity simulation

The metabolic state of the system was simulated with different levels of enzyme activities, for the three enzymes *PK*, *PGK* and *G6PDH*. To simulate changing activity in the enzyme module, the total enzyme concentration was multiplied by a certain fraction. To simulate changing enzyme activity in simplified rate laws, the rate law equation was multiplied by a certain fraction. After changing enzyme activities, the new steady state was obtained by simulating the system for a long enough time. The metabolite concentrations and associated metabolic states (e.g., inhibited hemoglobin level) were compared across rate laws and verified against physiological studies. All simulations were performed on the model constructed based on Mulquiney et al [34].

### Iterative substitution of approximate rate laws in place of enzyme modules

We started with the model constructed based on Mulquiney et al [34] (containing 22 enzyme modules) and iteratively replaced the modules with four different simplified rate laws. We iteratively increased the number of modules replaced by rate laws, at intervals of 1, 2, 3, 6, 9, 12, 15, 18 and 22. Together with the original model consisting entirely of enzyme modules, we built a total of 37 models with different rate laws. We then performed 18 different perturbations on those models. The perturbations fell into three main categories: local metabolite perturbations where change of metabolite concentration is less than 10 %, non-linear metabolite perturbations where change of metabolite concentration is greater than 10 %, perturbations through rate constant where the rate constant of a particular reaction was altered. The specific perturbation names can be found in Additional file 1: Figure S3. Models with replaced rate laws were compared against model containing all enzyme modules through correlation and percent error in metabolite RT and MP.

### Single module replacement

To test the effect of replacing single module on the network dynamics, we started with the model constructed based on Mulquiney et al [34] (containing 22 enzyme modules) and built 22 different models by replacing each of the enzyme modules with mass action kinetics in a single model. We then compared those 22 models against the original model consisting entirely of enzyme modules through correlation of metabolite RT across 18 different perturbations. We ranked each model based on its metabolite RT correlation with the original model in a perturbation. We then summed up the rank scores for each model across 18 different perturbations to obtain their final rank score. Lower rank score meant less change in dynamic response when the module is replaced with mass action kinetics. We compared the final rank against two factors that could determine the impact of simplified rate law replacing the enzyme module. One factor is reaction thermodynamic irreversibility, which is calculated as (reaction equilibrium constant - mass action ratio)/reaction equilibrium constant. The other is the largest metabolite concentration in the reaction.

### Parameter sampling

We used the model constructed by Bordbar et al [33] for parameter sampling. The range of metabolite concentrations were based on the physiologically measured concentrations from 24 healthy individuals [33]. For unmeasured metabolites whose concentrations were taken from literature, their range was set based on the average standard error of measured metabolite concentrations. The sampled metabolite concentrations were constrained by the second law of thermodynamics, where equilibrium constants of the reaction were derived from eQuilibrator [39, 40]. We then used gpSampler in cobratoolbox to obtain 1000 sets of metabolite concentrations that fell in the physiologically relevant range and satisfied the thermodynamic constraint [35, 41]. The sampled fluxes of the model were obtained directly from Bordbar et al [33]. The rate constants of the reactions were then calculated from equilibrium constants, sampled metabolite concentrations and sampled fluxes. As a result, a total of 300 models were constructed from the sampled parameters, concentrations and fluxes.

To compare the dynamic behavior of models with different sets of parameters, concentrations and fluxes, we performed three different perturbations on the 300 sampled models. The three perturbations were: changing ATP, ADP, Pi concentrations, changing NAD/NADH concentrations and changing FDP concentration. It was worth noting that only 63 models were able to achieve stable steady states after the perturbations. The RT and MP of the metabolites in those models were calculated from the perturbation profiles. We then selected metabolites with MP over 5 % and compared the dynamic response across models.

### Physiological simulation

We used the model constructed based on Mulquiney et al [34] (containing 22 enzyme modules) for physiological simulation. The physiological condition we chose was the hypoxia state of erythrocytes, and we simulated such a state by changing the external concentration of oxygen to 30 % of its original level. Due to the known role of Band III (BIII) protein in erythrocytes under hypoxia condition, we added binding reactions of BIII to hemoglobin, *PFK*, *GAPDH* and *ALD* [37]. We replaced the rest of the modules with different approximate rate laws, simulated the models under hypoxia condition for long enough time until steady state was reached, and compared the time profiles of metabolites across rate laws.

## Abbreviations

13DPG, 3-Phospho-D-glyceroyl phosphate; 23DPG, 2,3-diphosphoglycerate; 2PG, 2-Phospho-D-glycerate; 3PG, 3-Phospho-D-glycerate; 6PGC, 6-Phospho-D-gluconate; 6PGL, 6-phospho- D-glucono-1,5-lactone; *ADK*, adenylate kinase; ADP, adenosine diphosphate; *ALD*, aldolase; AMP, adenosine monophosphate; ATP, adenosine triphosphate; CO2, carbon dioxide; DHAP, dihydroxyacetone phosphate; *DPGASE*, bisphosphoglycerate phosphatase; *DPGM*, bisphosphoglycerate mutase; *ENO*, enolase; F6P, D-Fructose 6-phosphate; FDP, D-Fructose 1,6-bisphosphate; G3P, glyceraldehyde 3-phosphate; G6P, D-Glucose 6-phosphate; *G6PDH*, glucose 6-phosphate dehydrogenase; *GAPDH*, glyceraldehyde 3-phosphate dehydrogenase; GLC-D, D-Glucose; *GND*, phosphogluconate dehydrogenase; *GSSGR*, glutathione reductase; H+, hydrogen ion; *HEX1*, hexokinase; LAC-L, L-Lactate; *LDH*, lactate dehydrogenase; MAN6P, D-Mannose 6-phosphate; MP, maximum perturbation; NAD, Nicotinamide adenine dinucleotide; NADH, Nicotinamide adenine dinucleotide - reduced; NADP, nicotinamide adenine dinucleotide phosphate; NADPH, nicotinamide adenine dinucleotide phosphate - reduced; O2, oxygen; PEP, phosphoenolpyruvate; *PFK*, phosphofructokinase; *PGI*, glucose 6-phosphate isomerase; *PGK*, phosphoglycerate kinase; *PGLASE*, 6-phosphogluconolactonase; *PGLM*, phosphoglycerate mutase; Pi, orthophosphate; *PK*, pyruvate kinase; PRPP, 5-Phospho-alpha-D-ribose 1-diphosphate; PYR, pyruvate; QSS, quasi-steady state; R5P, alpha-D-Ribose 5-phosphate; *R5PE*, ribulose 5-phosphate epimerase; *R5PI*, ribulose 5-phosphate isomerase; RBC, red blood cell; RT, relaxation time; RU5P-D, D-Ribulose 5-phosphate; *TALA*, transaldolase; *TKI*/*TKII*, transketolase; *TPI*, triose phosphate isomerase

## Additional file

Additional file 1: Additional simulations performed for rate law comparison and data used for enzyme module construction. (DOCX 5388 kb)
