# Supplementary material for: Evaluation of rate law approximations in bottom-up kinetic models of metabolism
Source: BMC Syst Biol. 2016 Jun 6;10:40. doi: 10.1186/s12918-016-0283-2 (PMC4895898; doi:10.1186/s12918-016-0283-2)
Supplement: Additional file 1: — Additional simulations performed for rate law comparison and data used for enzyme module construction. (DOCX 5388 kb) [file 12918_2016_283_MOESM1_ESM.docx]

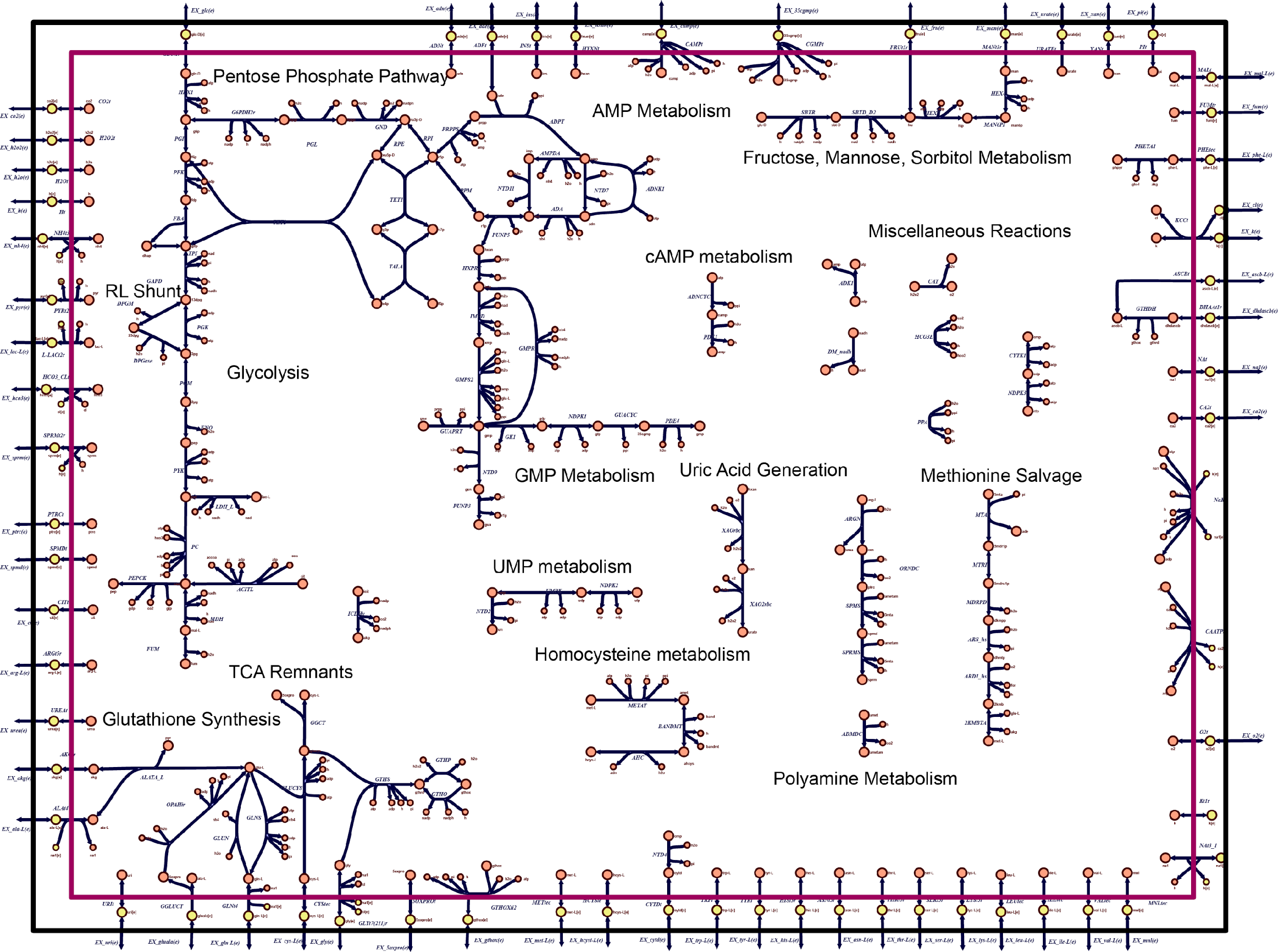


Figure S1. Detailed pathway map for the whole-cell kinetic model of erythrocyte [[1]](https://paperpile.com/c/dMwSsT/Oyq8). The model is available upon request.


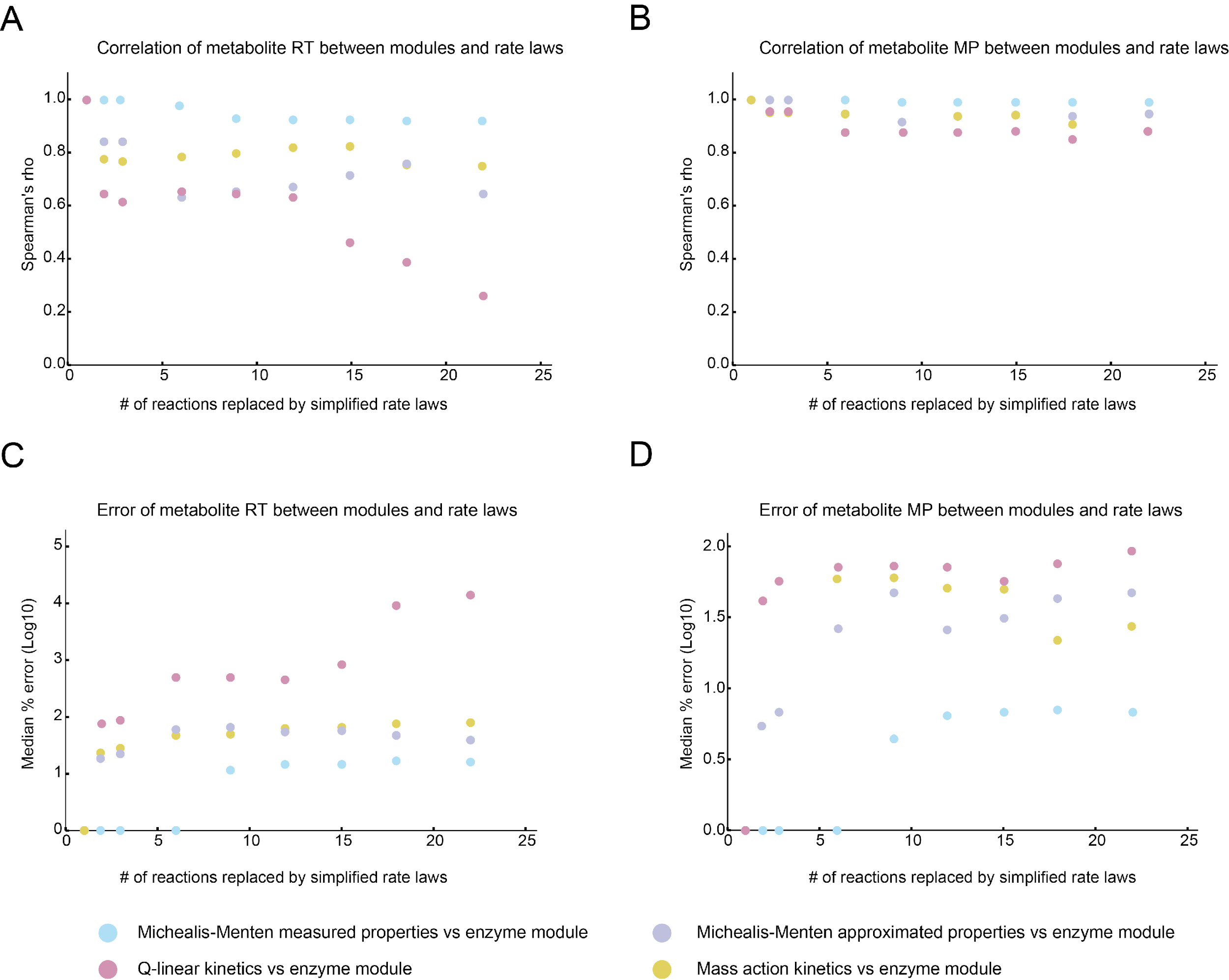


Figure S2. Iterative replacement of enzyme modules by simplified rate laws. Various numbers of enzyme modules were replaced by Michaelis-Menten rate law with measured properties, Michaelis-Menten rate law with approximated properties, Q-linear kinetics and mass action kinetics respectively, as annotated with different colors. The perturbation performed was decreasing R5P concentration by 10% in the model at time 0. The models were then simulated with long enough time until reaching steady state. The correlation and median percent error of metabolite RT and MP between model with enzyme modules and model with replaced rate laws were calculated. All simulations were performed on the model constructed based on Mulquiney et al [[2]](https://paperpile.com/c/dMwSsT/1iOm). We built the model full of enzyme modules and also replaced the modules with different simplified rate laws. A) Correlation of metabolite RT between modules and replaced rate laws. B) Correlation of metabolite MP between modules and replaced rate laws. C) Median percent error of metabolite RT between modules and replaced rate laws. D) Median percent error of metabolite MP between modules and replaced rate laws. Spearman’s rho: Spearman’s rank correlation coefficient.


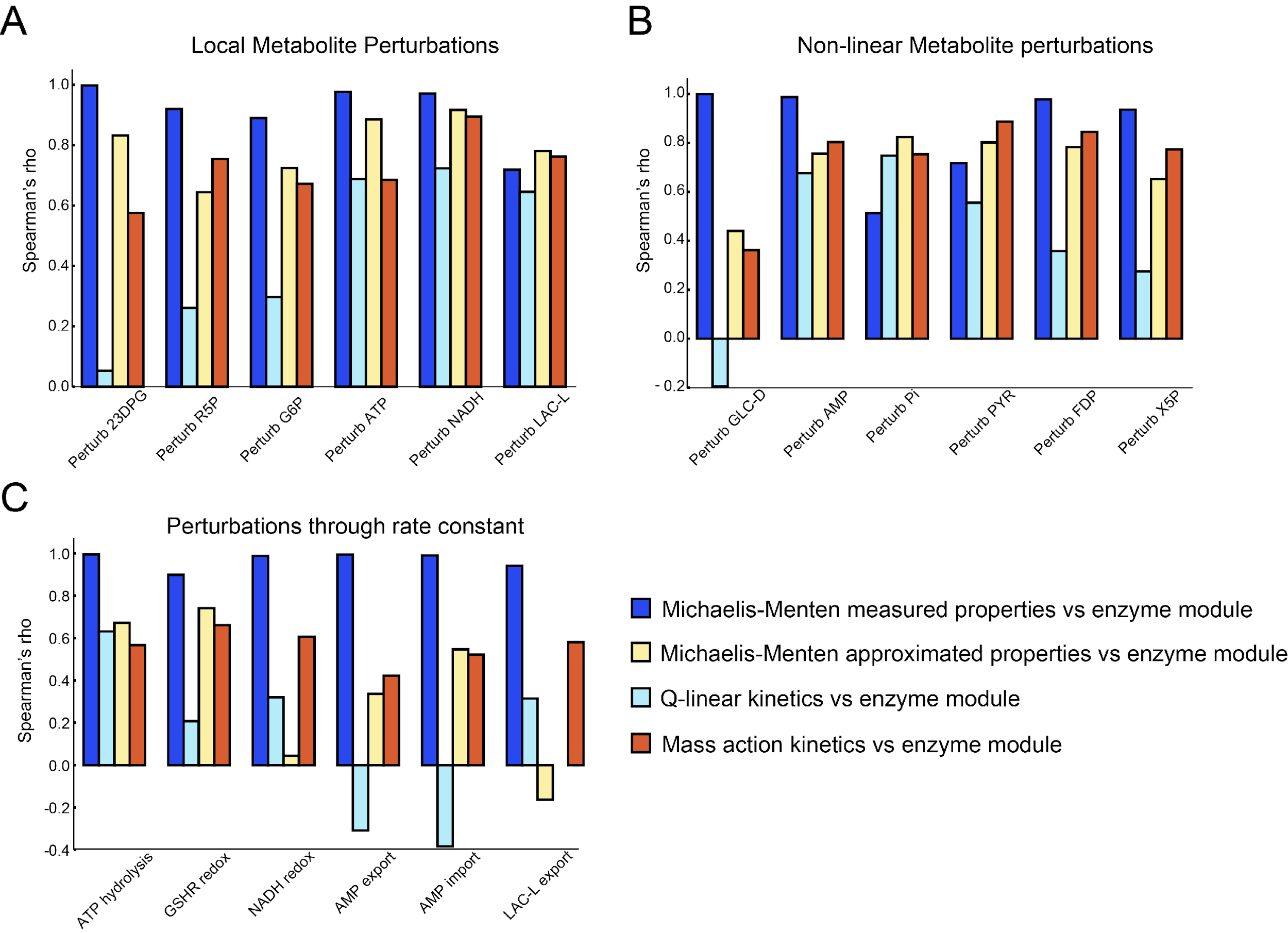


Figure S3. Comparison of four simplified rate laws against the enzyme module by metabolite RT. The response of metabolites under different perturbations was compared between model with all simplified rate laws and model with all enzyme modules. Multiple perturbations were performed and listed as three main categories: local metabolite perturbations where the change of metabolite concentration is less than 10%, non-linear metabolite perturbations where the change of metabolite concentration is greater than 10%, perturbations through rate constant where the rate constant of a particular reaction was altered. All simulations were performed on the model constructed based on Mulquiney et al [[2]](https://paperpile.com/c/dMwSsT/1iOm). We built the model full of enzyme modules and also replaced the modules with different simplified rate laws. A) Correlation of metabolite RT between rate laws and enzyme module for local metabolite perturbations. B) Correlation of metabolite RT between rate laws and enzyme module for non-linear metabolite perturbations. C) Correlation of metabolite RT between rate laws and enzyme module for perturbations through rate constant. Spearman’s rho: Spearman’s rank correlation coefficient.


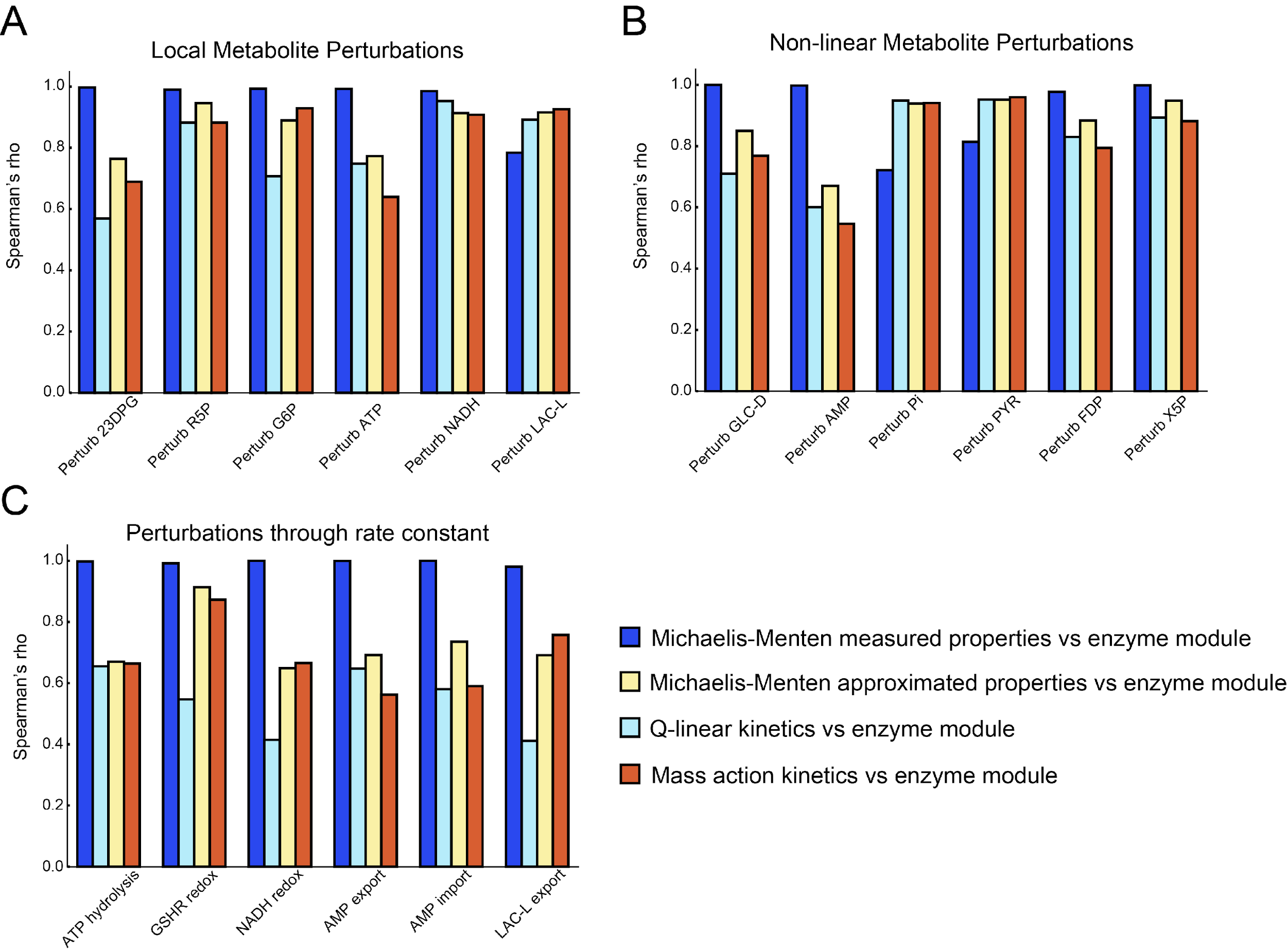


Figure S4. Comparison of four simplified rate laws against the enzyme module by metabolite MP. The response of metabolites under different perturbations was compared between model with all simplified rate laws and model with all enzyme modules. Multiple perturbations were performed and listed as three main categories: local metabolite perturbations where change of metabolite concentration is less than 10%, non-linear metabolite perturbations where change of metabolite concentration is greater than 10%, perturbations through rate constant where the rate constant of a particular reaction was altered. All simulations were performed on the model constructed based on Mulquiney et al [[2]](https://paperpile.com/c/dMwSsT/1iOm). We built the model full of enzyme modules and also replaced the modules with different simplified rate laws. A) Correlation of metabolite MP between rate laws and enzyme module for local metabolite perturbations. B) Correlation of metabolite MP between rate laws and enzyme module for non-linear metabolite perturbations. C) Correlation of metabolite MP between rate laws and enzyme module for perturbations through rate constant. Spearman’s rho: Spearman’s rank correlation coefficient.


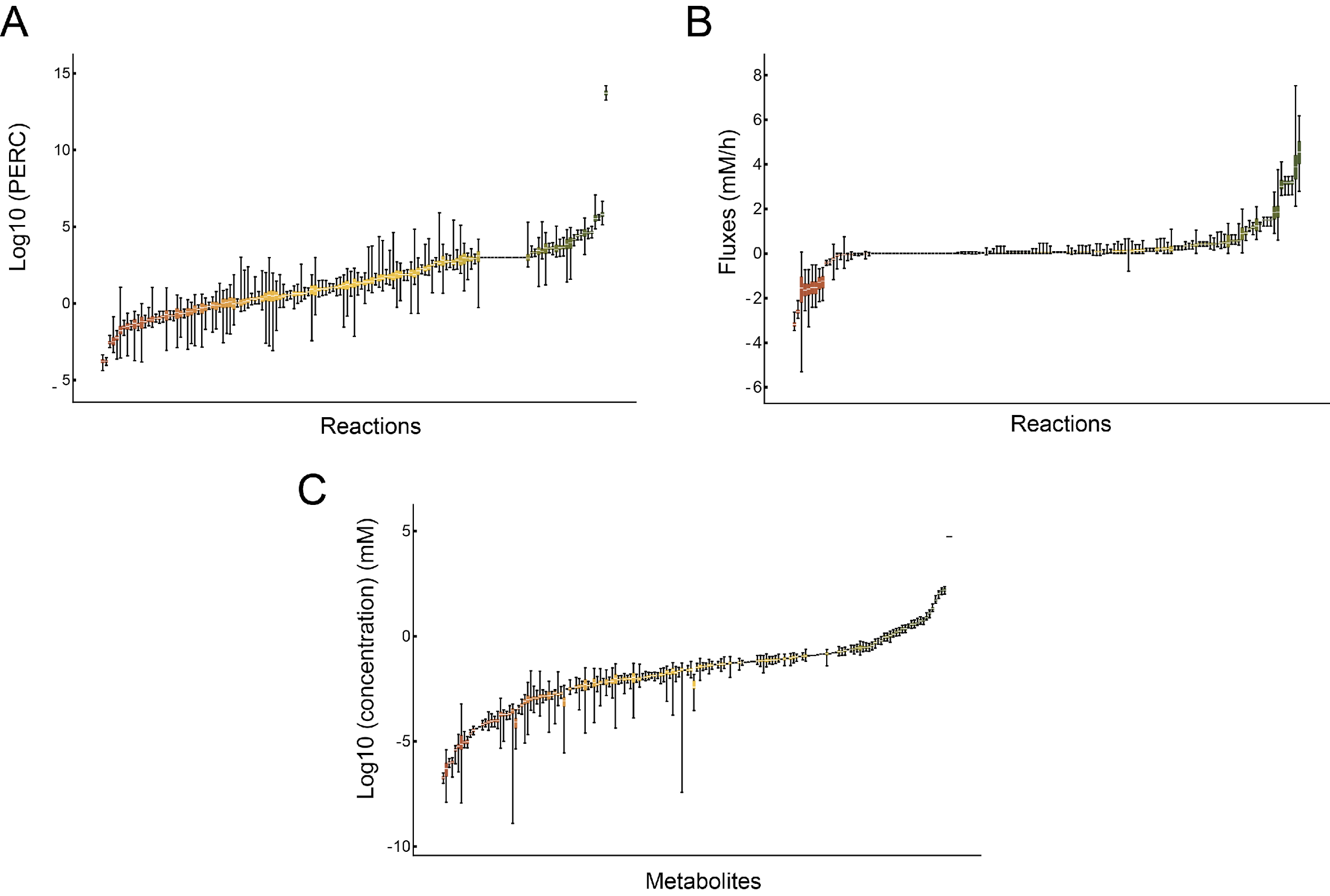


Figure S5. Sampled values of parameters, fluxes and metabolite concentrations for 300 models with mass action kinetics. The fluxes and metabolite concentrations were sampled within the physiologically-relevant range. Multiple reactions have zero fluxes and do not connect with the rest of the network. As a result, their fluxes and parameters were not sampled and shown as a flat line. A) Range of sampled pseudo-elementary rate constants (PERC) for all reactions in the model. B) Range of sampled fluxes for all reactions in the model. C) Range of sampled metabolite concentrations in the model. Based on mass action kinetics, the rate constant in the forward direction of a reaction is termed pseudo-elementary rate constant in the model. The sampling was done on the whole-cell kinetic model of erythrocyte constructed by Bordbar et al [[1]](https://paperpile.com/c/dMwSsT/Oyq8).


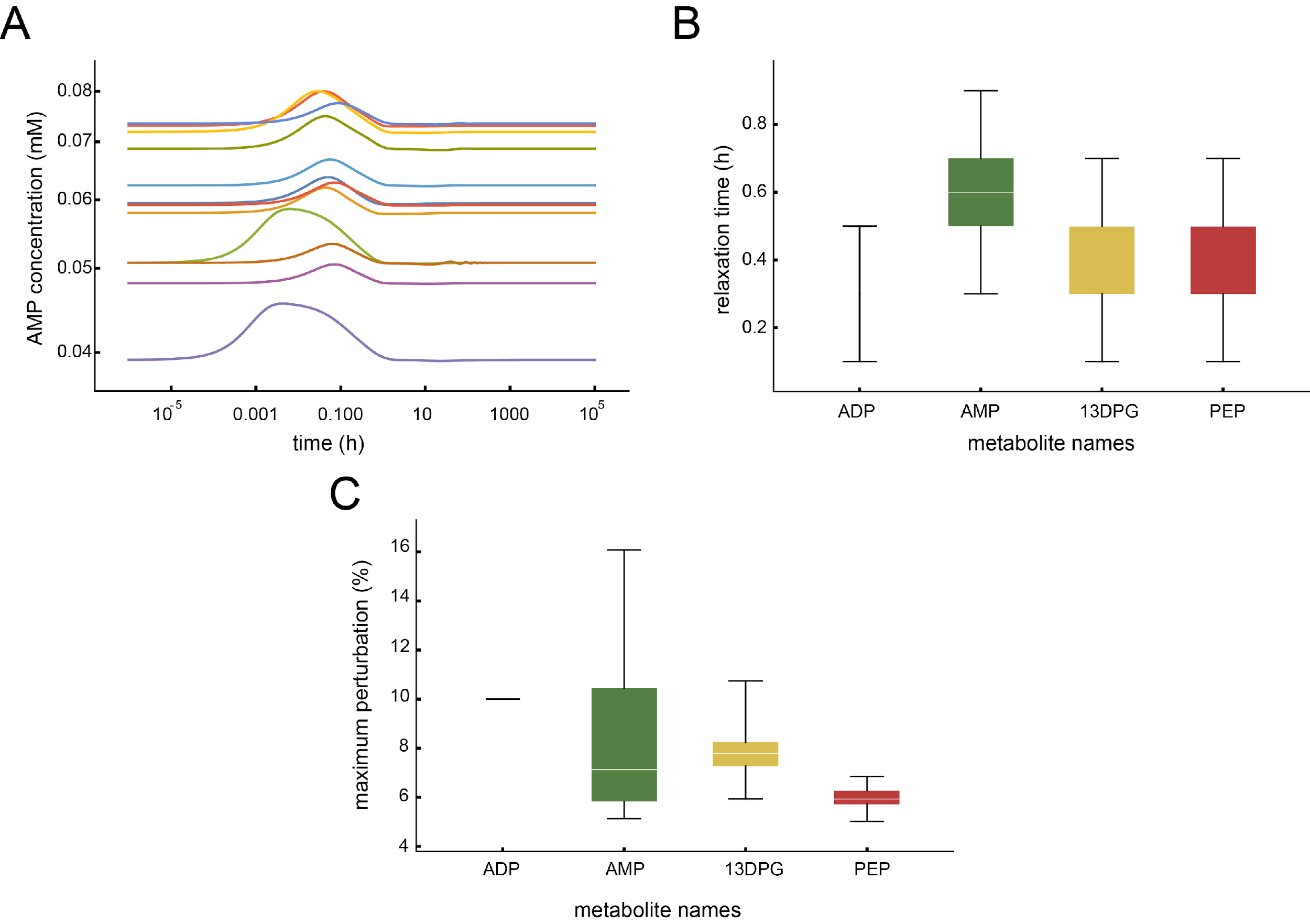


Figure S6. Response of sampled models against ATP/ADP/Pi perturbation. The concentrations of ATP, ADP and Pi were changed by the same amount at time 0 and the models were simulated long enough to reach steady state. Metabolites with maximum perturbation greater than 5% were selected and plotted. The concentration of ADP was changed by 10% across models and thus the percent of maximum perturbation shown was a flat line. A) AMP concentration change in response to ATP/ADP/Pi perturbation across models. B) Distribution of relaxation time across models for ADP, AMP, 13DPG and PEP in response to perturbation. C) Distribution of maximum perturbation across models for ADP, AMP, 13DPG and PEP in response to perturbation. The sampling and simulation were done on the whole-cell kinetic model of erythrocyte constructed by Bordbar et al [[1]](https://paperpile.com/c/dMwSsT/Oyq8). The models were sampled based on the fluxes and concentrations within the physiologically-relevant range.


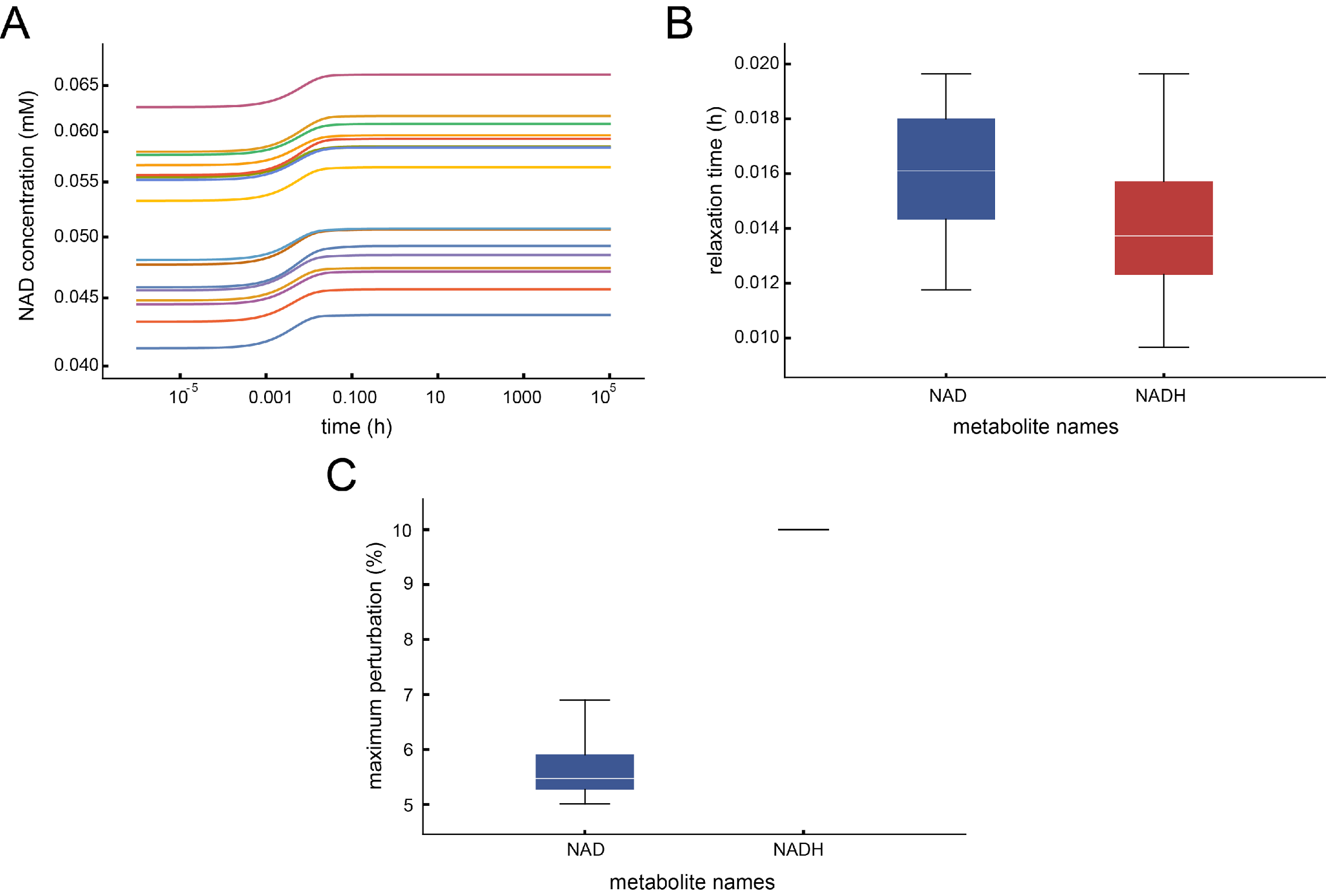


Figure S7. Response of sampled models against NAD/NADH perturbation. The concentrations of NAD and NADH were changed by the same amount at time 0 and the models were simulated long enough to reach steady state. Metabolites with maximum perturbation greater than 5% were selected and plotted. The concentration of NADH was changed by 10% across models and thus percent of maximum perturbation shown was a flat line. A) NAD concentration change in response to NAD/NADH perturbation across models. B) Distribution of relaxation time across models for NAD and NADH in response to perturbation. C) Distribution of maximum perturbation across models for NAD and NADH in response to perturbation. The sampling and simulation were done on the whole-cell kinetic model of erythrocyte constructed by Bordbar et al [[1]](https://paperpile.com/c/dMwSsT/Oyq8). The models were sampled based on the fluxes and concentrations within the physiologically-relevant range.


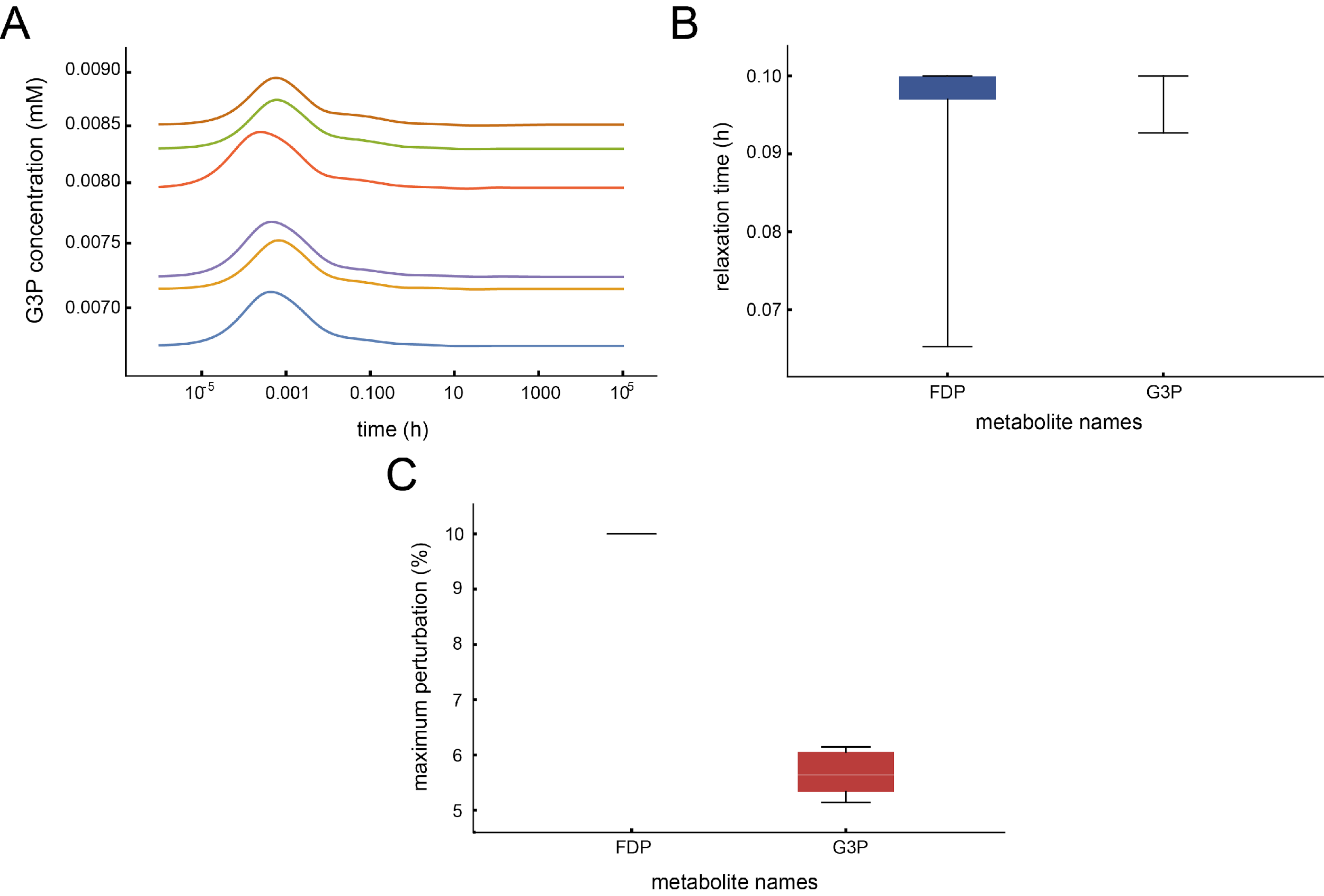


Figure S8. Response of sampled models against FDP perturbation. The concentration of FDP was changed by 10% at time 0 and the models were simulated long enough to reach steady state. Metabolites with maximum perturbation greater than 5% were selected and plotted. The concentration of FDP was changed by 10% across models and thus percent of maximum perturbation shown was a flat line. A) G3P concentration change in response to FDP perturbation across models. B) Distribution of relaxation time across models for FDP and G3P in response to perturbation. C) Distribution of maximum perturbation across models for FDP and G3P in response to perturbation. The sampling and simulation were done on the whole-cell kinetic model of erythrocyte constructed by Bordbar et al [[1]](https://paperpile.com/c/dMwSsT/Oyq8). The models were sampled based on the fluxes and concentrations within the physiologically-relevant range.


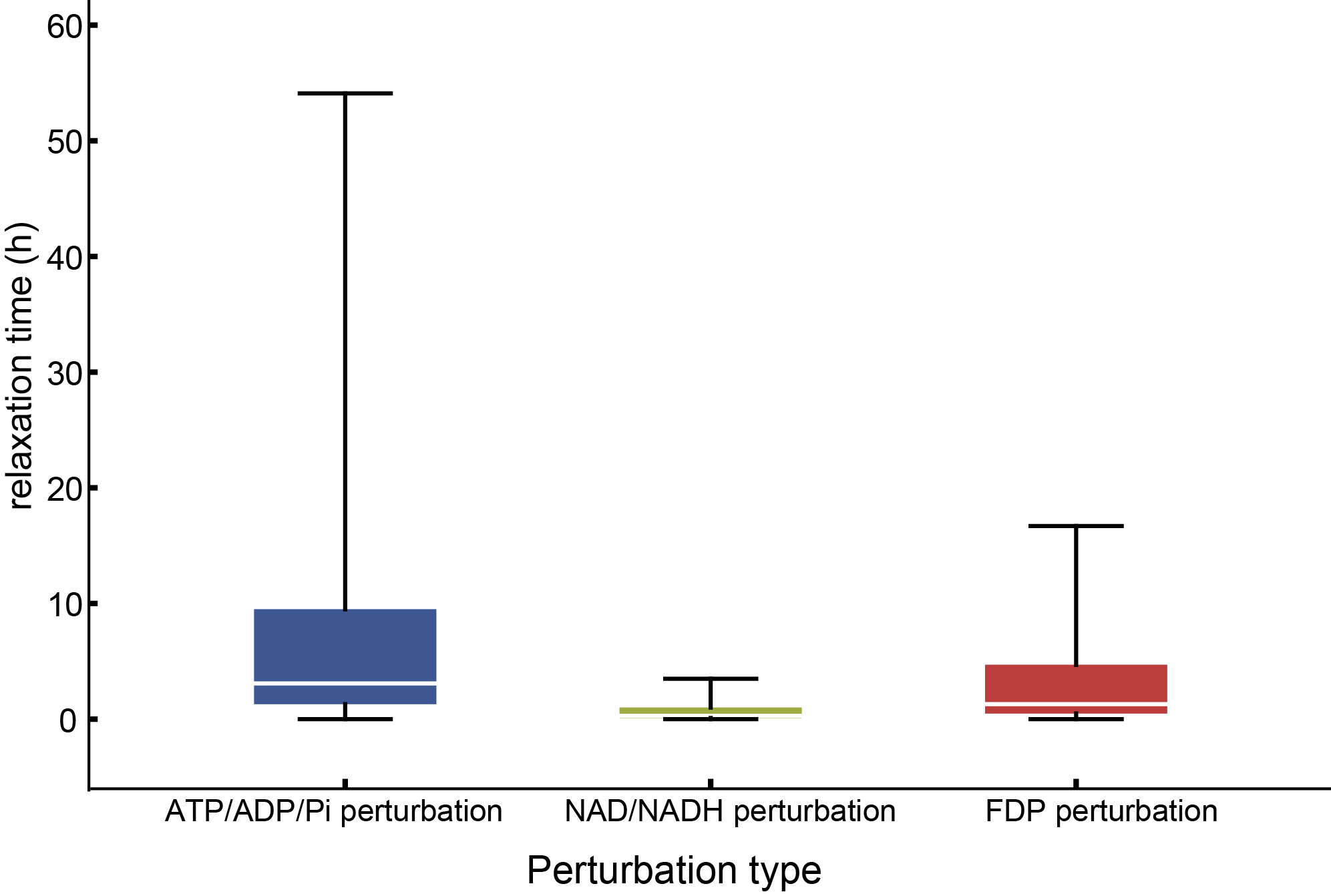


Figure S9. Distribution of relaxation time for all metabolites in one sampled model against different perturbations. The distributions of relaxation time for all metabolites against ATP/ADP/Pi perturbation, NAD/NADH perturbation, FPD perturbation in one sampled model were shown. Compared to the relaxation time range for all metabolites, the relaxation time range for single metabolite across models was much smaller in all three perturbations shown (Figure S3-5). The sampling and simulation were done on the whole-cell kinetic model of erythrocyte constructed by Bordbar et al [[1]](https://paperpile.com/c/dMwSsT/Oyq8). The models were sampled based on the fluxes and concentrations within the physiologically-relevant range.


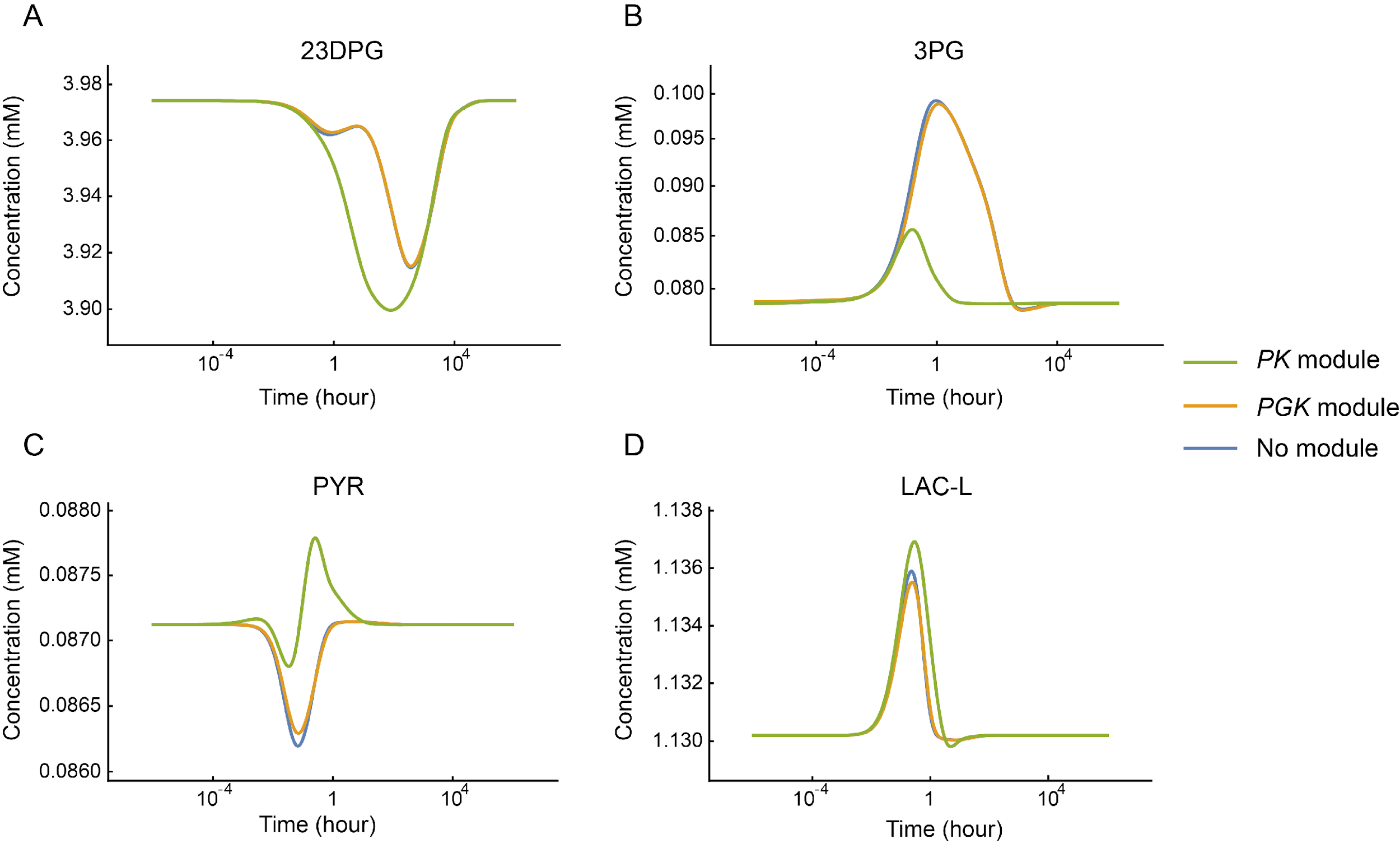


Figure S10. The dynamic response of metabolites following ATP hydrolysis perturbation. The response of four different metabolites for three versions of the model (no module, an addition of the *PK* module, and an addition of the *PGK* module) are shown. A) Dynamic response of 23DPG. B) Dynamic response of 3PG. C) Dynamic response of PYR. D) Dynamic response of LAC-L. No obvious trend can be found for how the dynamics of metabolites change upon single module addition. For example, the addition of *PK* module caused a large change in the dynamics of 23DPG, 3PG and PYR while the addition of *PGK* module had little effect. The simulations were done on the whole-cell kinetic model of erythrocyte constructed by Bordbar et al [[1]](https://paperpile.com/c/dMwSsT/Oyq8).

Table S1. Change in metabolite RT and MP upon the addition of *PGK* or *PK* module

| Percent change in metabolite RT/MP compared to no module | Addition of the *PGK* module | Addition of the *PK* module |
| --- | --- | --- |
| 23DPG | 4.97 / -0.80 | -19.4 / 25.3 |
| 3PG | -2.73 / -1.84 | -98.6 / -68.4 |
| PYR | 40.0 / -10.4 | 1600 / -29.5 |
| LAC-L | 18.2 / -4.75 | 491 / 23.7 |


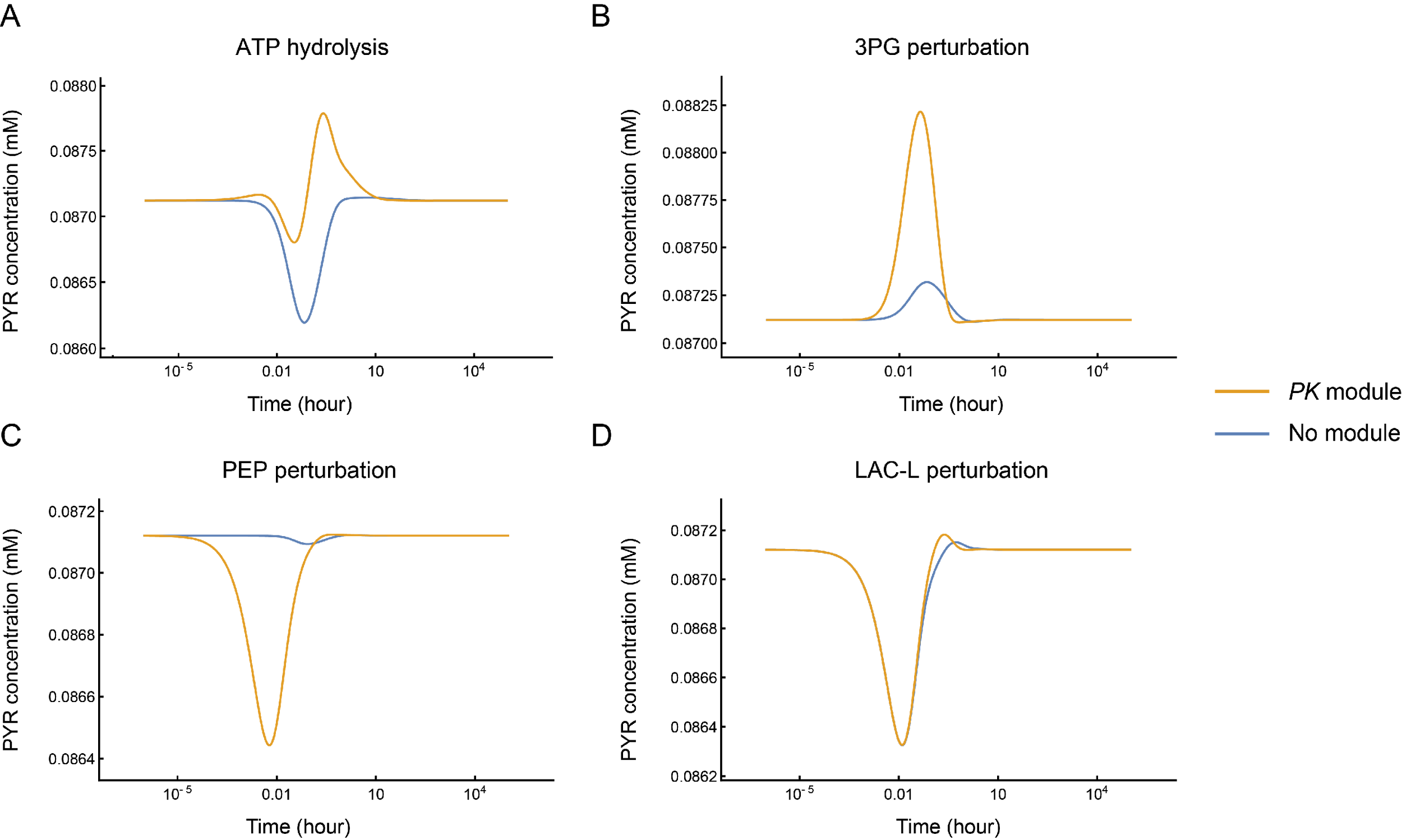


Figure S11. The dynamic response of PYR with and without the *PK* module under different perturbations. A) ATP hydrolysis. B) 3PG perturbation. C) PEP perturbation. D) LAC-L perturbation. For the same metabolite PYR, no consistent change in its dynamic response can be found after adding the *PK* module. The simulations were done on the whole-cell kinetic model of erythrocyte constructed by Bordbar et al [[1]](https://paperpile.com/c/dMwSsT/Oyq8).

Table S2. Change in relaxation time (RT) and maximum perturbation (MP) of PYR upon the addition of the *PK* module

| Perturbations | Percent change in RT/MP of PYR under perturbations after addition of the *PK* module |
| --- | --- |
| ATP hydrolysis | 3583.3/ -78.0 |
| 3PG perturbation | -42.9/ 324.6 |
| PEP perturbation | -71.4/ 754.4 |
| LAC-L perturbation | 400/ -0.45 |


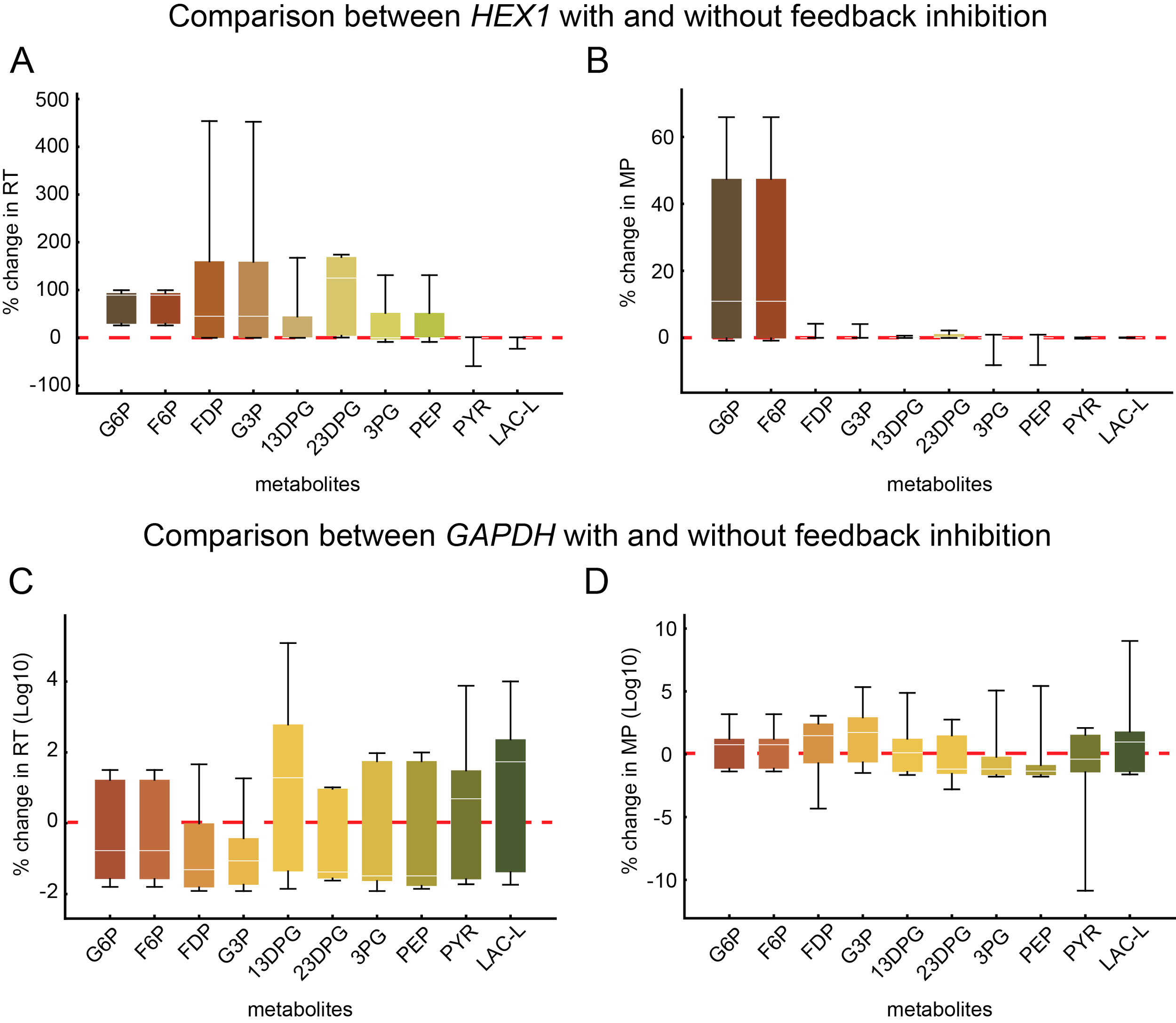


Figure S12. The effect of feedback inhibition on the relaxation time and maximum perturbation of the metabolites. A) The effect of *HEX1* feedback inhibition on RT of metabolites. B) The effect of *HEX1* feedback inhibition on MP of metabolites. C) The effect of *GAPDH* feedback inhibition on RT of metabolites. D) The effect of *GAPDH* feedback inhibition on MP of metabolites. The perturbations performed included ATP hydrolysis, NAD and NADH perturbation, FDP perturbation, G3P perturbation, 13DPG perturbation, 23DPG perturbation, 3PG perturbation, PEP perturbation, PYR perturbation, LAC-L perturbation. The simulations were done on the whole-cell kinetic model of erythrocyte constructed by Bordbar et al [[1]](https://paperpile.com/c/dMwSsT/Oyq8).


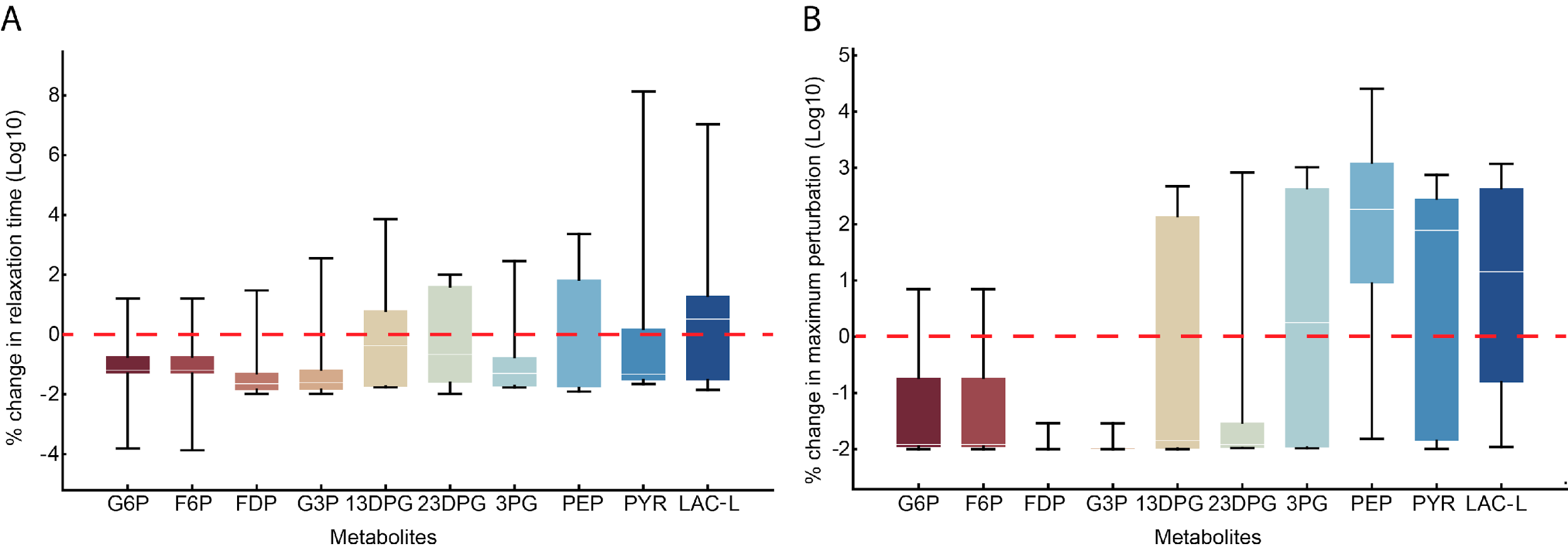


Figure S13. The effect of feedforward activation on the *PK* module with FDP as the effector molecule. A) The effect of feedforward activation on RT of the metabolites. B) The effect of feedforward activation on MP of the metabolites. The perturbations performed include G6P perturbation, F6P perturbation, FDP perturbation, G3P perturbation, 13DPG perturbation, 23DPG perturbation, 3PG perturbation, PEP perturbation, PYR perturbation, LAC-L perturbation. The simulations were done on the whole-cell kinetic model of erythrocyte constructed by Bordbar et al [[1]](https://paperpile.com/c/dMwSsT/Oyq8).


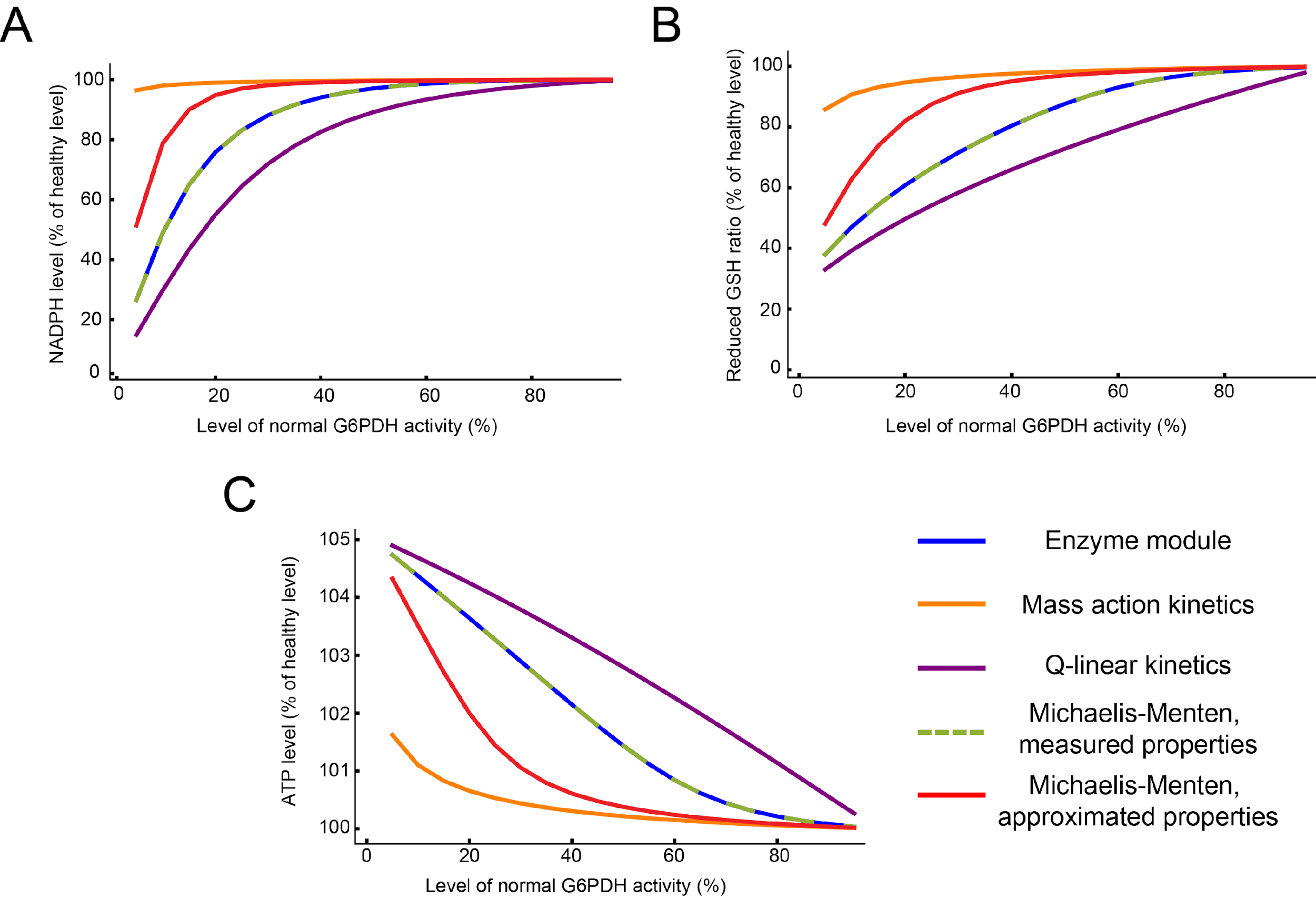


Figure S14. Comparison of model response at various levels of *G6PDH* activity across enzyme module and different rate laws. The normal enzyme activity is denoted as 100%. At different levels of enzyme activity, the steady state metabolite concentrations relative to its original level (at 100% enzyme activity) were shown. Reduced GSH ratio is the ratio of reduced glutathione over the sum of reduced and oxidized glutathione. All simulations were performed on the model constructed based on Mulquiney et al [[2]](https://paperpile.com/c/dMwSsT/1iOm). We built the model full of enzyme modules and also replaced the modules with different simplified rate laws. A) Relative NADPH concentration at different levels of *G6PDH* activity. B) Relative reduced GSH ratio at different levels of *G6PDH* activity. C) Relative ATP concentration at different levels of *G6PDH* activity.


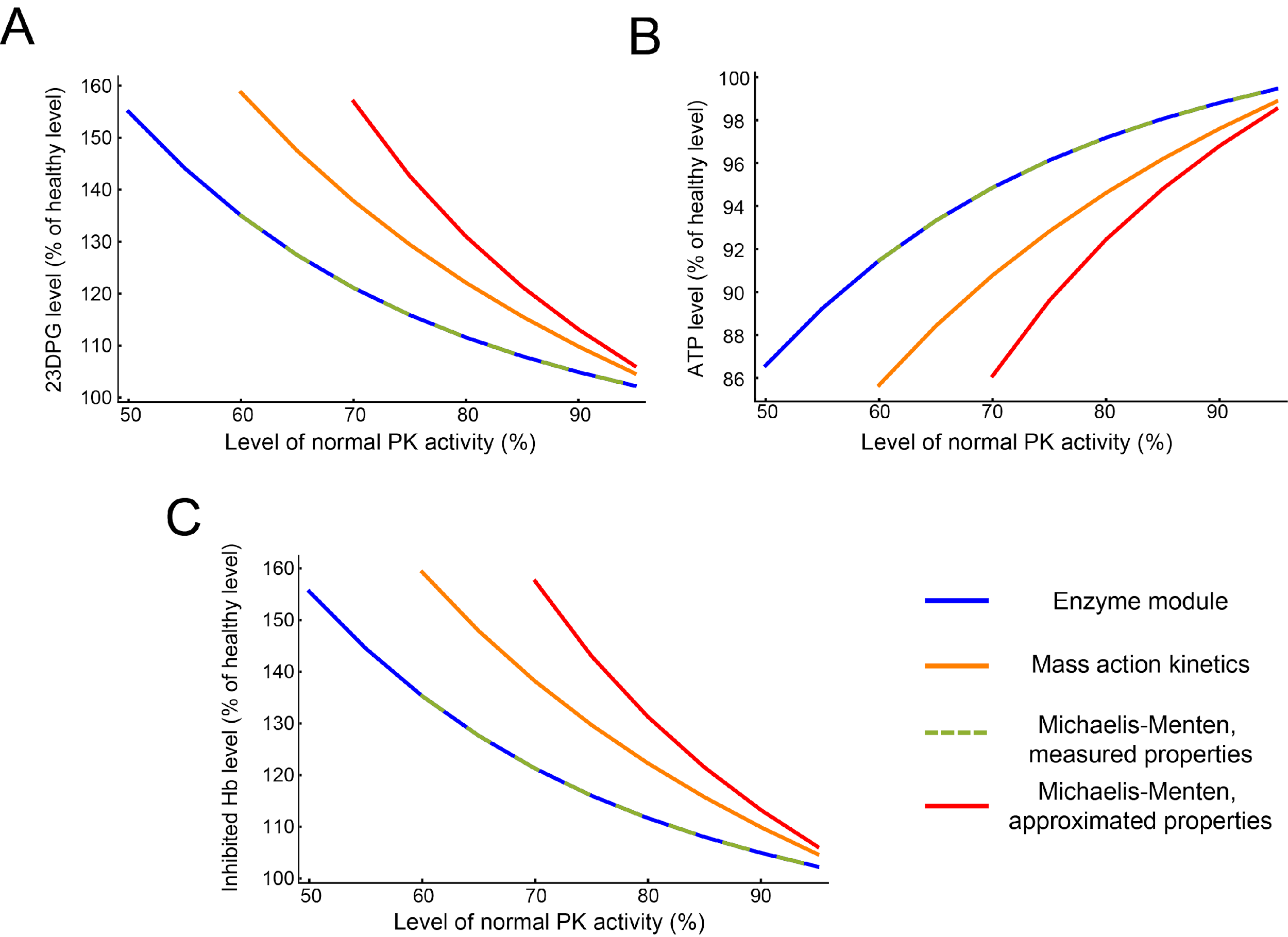


Figure S15. Comparison of model response at various levels of *PK* activity across enzyme module and different rate laws. The normal enzyme activity is denoted as 100%. At different levels of enzyme activity, the steady state metabolite concentrations relative to its original level (at 100% enzyme activity) were shown. We were not able to obtain steady state metabolite concentrations with enzyme activity change for Q-linear kinetics since the model was not able to reach a steady state even with 99% *PK* activity. Inhibited Hb level is the ratio of hemoglobin concentration bound with 23DPG over total hemoglobin concentration. All simulations were performed on the model constructed based on Mulquiney et al [[2]](https://paperpile.com/c/dMwSsT/1iOm). We built the model full of enzyme modules and also replaced the modules with different simplified rate laws. A) Relative 23DPG concentration at different levels of *PK* activity. B) Relative ATP concentration at different levels of *PK* activity. C) Relative inhibited Hb level at different levels of *PK* activity.


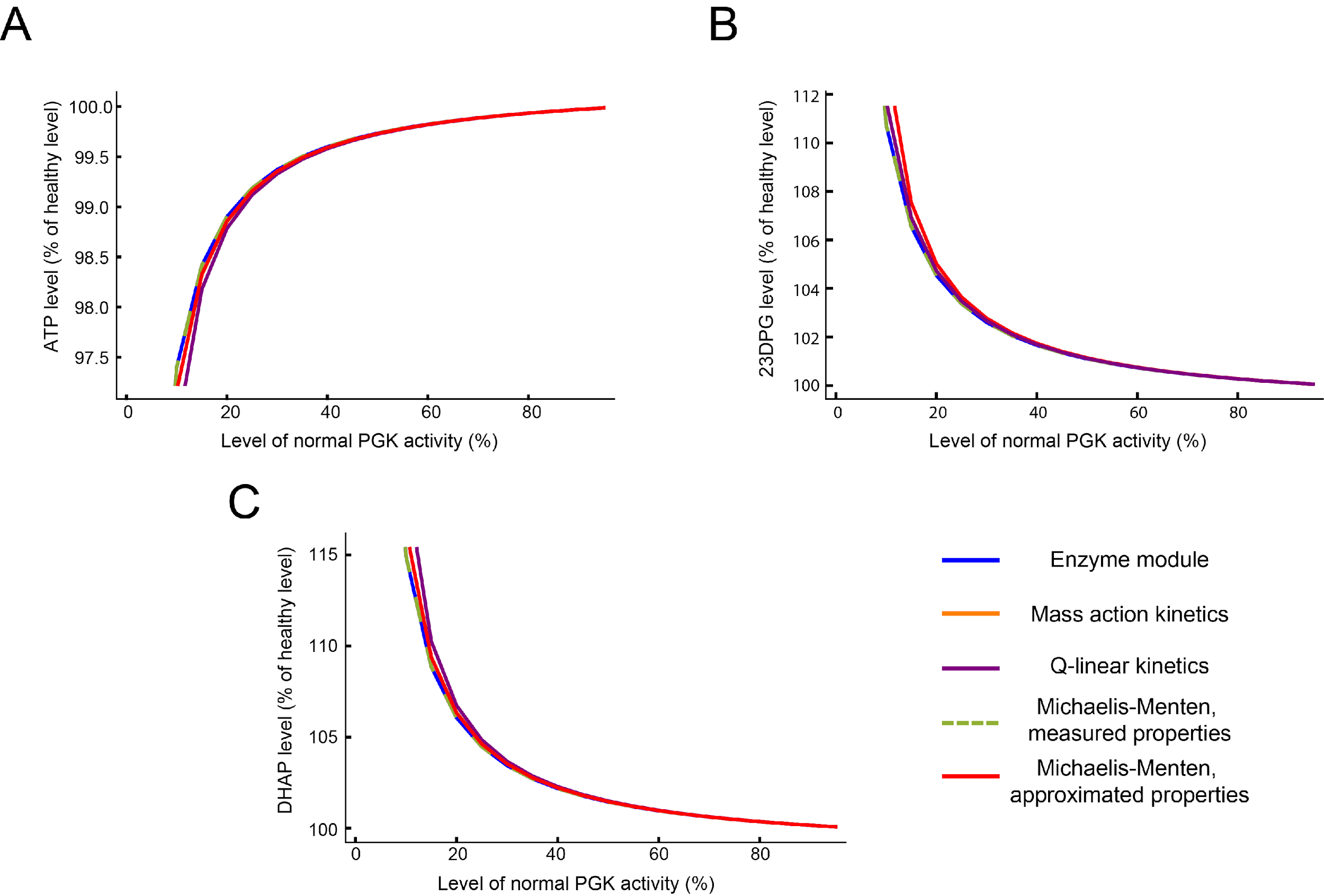


Figure S16. Comparison of model response at various levels of *PGK* activity across enzyme module and different rate laws. The normal enzyme activity is denoted as 100%. At different levels of enzyme activity, the steady state metabolite concentrations relative to its original level (at 100% enzyme activity) were shown. All simulations were performed on the model constructed based on Mulquiney et al [[2]](https://paperpile.com/c/dMwSsT/1iOm). We built the model full of enzyme modules and also replaced the modules with different simplified rate laws. A) Relative ATP concentration at different levels of *PGK* activity. B) Relative 23DPG concentration at different levels of *PGK* activity. C) Relative DHAP concentration at different levels of *PGK* activity.


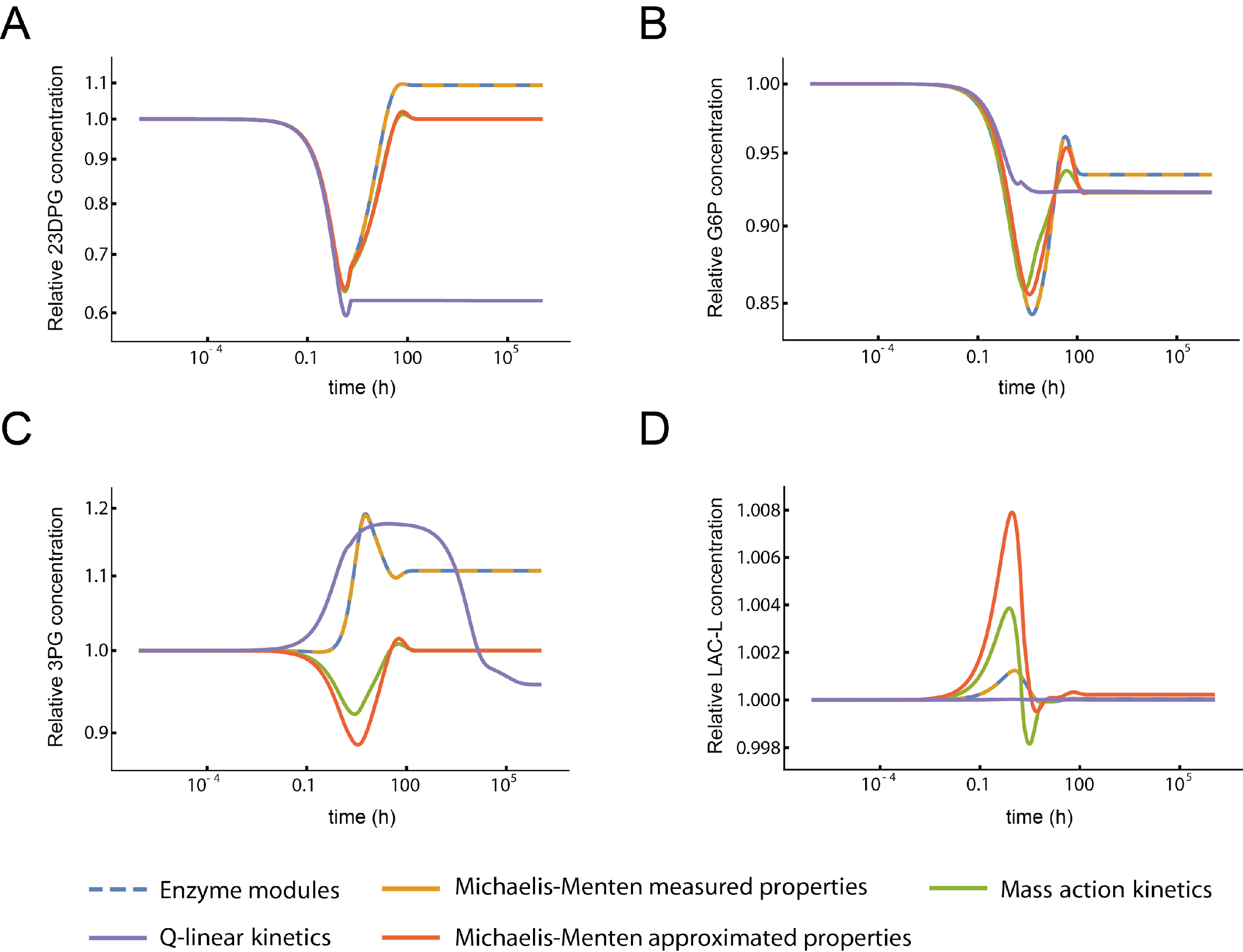


Figure S17. Comparison of erythrocyte response under hypoxia condition across enzyme module and different rate laws. The hypoxia condition was simulated by decreasing external oxygen concentration to 30% of its original level. The change of metabolite concentrations over time was compared across enzyme module and different rate laws. The metabolite response of model containing enzyme modules was also verified qualitatively against experimental data [[3]](https://paperpile.com/c/dMwSsT/tC3q). All simulations were performed on the model constructed based on Mulquiney et al [[2]](https://paperpile.com/c/dMwSsT/1iOm). We built the model full of enzyme modules and also replaced the modules with different simplified rate laws. A) Change of 23DPG concentration relative to its normal level. B) Change of G6P concentration relative to its normal level. C) Change of 3PG concentration relative to its normal level. D) Change of LAC-L concentration relative to its normal level.

Table S3. The parameters used in constructing the enzyme modules

| Enzyme | Reference | Parameter | Value | Unit | Source |
| --- | --- | --- | --- | --- | --- |
| *PGK* | [[4]](https://paperpile.com/c/dMwSsT/ceLM) | K_d-13DPG_ | 5.6 × 10^-8^ | M | human (exp. in E.coli) |
|  | [[4]](https://paperpile.com/c/dMwSsT/ceLM) | K_d-ADP_ | 2.9 × 10^-5^ | M | human (exp. in E.coli) |
|  | [[5]](https://paperpile.com/c/dMwSsT/0WQ0) | K_i-3PG_ | 1.7 × 10^-4^ | M | human RBC |
|  | [[6]](https://paperpile.com/c/dMwSsT/EAsF) | K_i-23DPG_ | 0.0024 | M | human RBC |
|  | [[7]](https://paperpile.com/c/dMwSsT/Z0Ag) | K_i-ATP-1_ | 2.3 × 10^-4^ | M | pig muscle |
|  | [[8]](https://paperpile.com/c/dMwSsT/dh6z) | K_i-ATP-2_ | 5.0 × 10^-4^ | M | human liver |
| *HEX1* | [[9]](https://paperpile.com/c/dMwSsT/fNy8) | K_d-GLC-D_ | 3.8 × 10^-5^ | M | human RBC |
|  | [[9]](https://paperpile.com/c/dMwSsT/fNy8) | K_d-ATP_ | 0.00206 | M | human RBC |
|  | [[6]](https://paperpile.com/c/dMwSsT/EAsF) | K_i-23DPG_ | 0.0055 | M | human RBC |
| *ADK* | [[10]](https://paperpile.com/c/dMwSsT/l88J) | K_d-AMP_ | 3.3 × 10^-4^ | M | human RBC |
|  | [[10]](https://paperpile.com/c/dMwSsT/l88J) | K_d-ATP_ | 9.5 × 10^-4^ | M | human RBC |
|  | [[10]](https://paperpile.com/c/dMwSsT/l88J) | K_d-ADP-1_ | 1.2 × 10^-4^ | M | human RBC |
|  | [[10]](https://paperpile.com/c/dMwSsT/l88J) | K_d-ADP-2_ | 2.2 × 10^-4^ | M | human RBC |
| *G6PDH* | [[11]](https://paperpile.com/c/dMwSsT/xAn7) | K_d-G6P_ | 2.7 × 10^-5^ | M | human RBC |
|  | [[11]](https://paperpile.com/c/dMwSsT/xAn7) | K_d-NADP_ | 1.9 × 10^-5^ | M | human RBC |
|  | [[12]](https://paperpile.com/c/dMwSsT/bTD8) | K_d-NADPH_ | 0.0105 | M | human RBC |
| *GAPDH* | [[13]](https://paperpile.com/c/dMwSsT/UYDn) | K_d-NAD_ | 4.5 × 10^-5^ | M | human RBC |
|  | [[13]](https://paperpile.com/c/dMwSsT/UYDn) | K_d-G3P_ | 3.1 × 10^-4^ | M | human RBC |
|  | [[13]](https://paperpile.com/c/dMwSsT/UYDn) | K_d-Pi_ | 7.8 × 10^-5^ | M | human RBC |
|  | [[14]](https://paperpile.com/c/dMwSsT/fGVn) | K_d-NADH_ | 1.3 × 10^-5^ | M | rabbit skeletal muscle |
|  | [[15]](https://paperpile.com/c/dMwSsT/mFm6) | K_d-13DPG_ | 8 × 10^-7^ | M | human RBC |
|  | [[14]](https://paperpile.com/c/dMwSsT/fGVn) | K_i-3PG_ | 2 × 10^-5^ | M | rabbit muscle |
|  | [[13]](https://paperpile.com/c/dMwSsT/UYDn) | K_i-G3P_ | 3.1 × 10^-4^ | M | human RBC |
| *PFK* | [[16]](https://paperpile.com/c/dMwSsT/EsUh) | K_d-ATP_ | 6.8 × 10^-5^ | M | rat RBC |
|  | [[16]](https://paperpile.com/c/dMwSsT/EsUh) | K_d-F6P_ | 7.5 × 10^-5^ | M | rat RBC |
|  | [[17]](https://paperpile.com/c/dMwSsT/9CVv) | K_i-ADP_ | 1.1 × 10^-4^ | M | rabbit muscle |
|  | [[18]](https://paperpile.com/c/dMwSsT/GosQ) | K_a-AMP_ | 3.5 × 10^-5^ | M | rat RBC |
| *PK* | [[19]](https://paperpile.com/c/dMwSsT/pJzv) | K_d-PEP_ | 2.25 × 10^-4^ | M | human RBC |
|  | [[19]](https://paperpile.com/c/dMwSsT/pJzv) | K_d-ADP_ | 4.74 × 10^-4^ | M | human RBC |
|  | [[20]](https://paperpile.com/c/dMwSsT/fSQq) | K_a-FDP_ | 5 × 10^-6^ | M | human RBC |
|  | [[19]](https://paperpile.com/c/dMwSsT/pJzv) | K_i-ATP_ | 3.39 × 10^-3^ | M | human RBC |
| *LDH* | [[2]](https://paperpile.com/c/dMwSsT/1iOm) | K_d-NAD_ | 1.07 × 10^-4^ | M | human RBC |
|  | [[21]](https://paperpile.com/c/dMwSsT/0RtE) | K_d-PYR_ | 1.01 × 10^-4^ | M | human RBC |
|  | [[21]](https://paperpile.com/c/dMwSsT/0RtE) | K_d-NADH_ | 6.8 × 10^-6^ | M | human RBC |
|  | [[22]](https://paperpile.com/c/dMwSsT/HDTb) | K_d-LAC-L_ | 0.007 | M | human RBC |
| *GND* | [[23]](https://paperpile.com/c/dMwSsT/Y6n7) | K_d-6PGC_ | 2 × 10^-5^ | M | human RBC |
|  | [[24]](https://paperpile.com/c/dMwSsT/u9pK) | K_d-NADP_ | 9.8 × 10^-6^ | M | sheep liver |
|  | [[25]](https://paperpile.com/c/dMwSsT/EB9N) | K_d-CO2_ | 1.5 × 10^-5^ | M | human RBC |
|  | [[25]](https://paperpile.com/c/dMwSsT/EB9N) | K_d-RU5P-D_ | 3 × 10^-5^ | M | human RBC |
|  | [[24]](https://paperpile.com/c/dMwSsT/u9pK) | K_d-NADPH_ | 5 × 10^-7^ | M | sheep liver |
|  | [[23]](https://paperpile.com/c/dMwSsT/Y6n7) | K_i-NADPH_ | 3 × 10^-5^ | M | human RBC |
| *PGI* | [[26]](https://paperpile.com/c/dMwSsT/Nt7U) | K_d-G6P_ | 0.85 | M | yeast |
|  | [[26]](https://paperpile.com/c/dMwSsT/Nt7U) | K_d-F6P_ | 0.117 | M | yeast |

Note: K_d_ is the dissociation constant of the enzyme form bound with metabolite. In cases where only K_m_ data is available, we approximated it as the K_d_ value. K_i_ is the inhibitor constant for the specific inhibitor on the enzyme form.

**References**

[1. Bordbar A, McCloskey D, Zielinski DC, Sonnenschein N, Jamshidi N, Palsson BO. Personalized Whole-Cell Kinetic Models of Metabolism for Discovery in Genomics and Pharmacodynamics. cels. Elsevier; 2015;1:283–92.](http://paperpile.com/b/dMwSsT/Oyq8)

[2. Mulquiney PJ, Kuchel PW. Model of 2,3-bisphosphoglycerate metabolism in the human erythrocyte based on detailed enzyme kinetic equations: equations and parameter refinement. Biochem. J. 1999;342 Pt 3:581–96.](http://paperpile.com/b/dMwSsT/1iOm)

[3. Kinoshita A, Tsukada K, Soga T, Hishiki T, Ueno Y, Nakayama Y, et al. Roles of hemoglobin Allostery in hypoxia-induced metabolic alterations in erythrocytes: simulation and its verification by metabolome analysis. J. Biol. Chem. 2007;282:10731–41.](http://paperpile.com/b/dMwSsT/tC3q)

[4. Flachner B, Varga A, Szabó J, Barna L, Hajdú I, Gyimesi G, et al. Substrate-assisted movement of the catalytic Lys 215 during domain closure: site-directed mutagenesis studies of human 3-phosphoglycerate kinase. Biochemistry. 2005;44:16853–65.](http://paperpile.com/b/dMwSsT/ceLM)

[5. Fermo E, Bianchi P, Chiarelli LR, Maggi M, Mandarà GML, Vercellati C, et al. A new variant of phosphoglycerate kinase deficiency (p.I371K) with multiple tissue involvement: molecular and functional characterization. Mol. Genet. Metab. 2012;106:455–61.](http://paperpile.com/b/dMwSsT/0WQ0)

[6. Ponce J, Roth S, Harkness DR. Kinetic studies on the inhibition of glycolytic kinases of human erythrocytes by 2,3-diphosphoglyceric acid. Biochim. Biophys. Acta. 1971;250:63–74.](http://paperpile.com/b/dMwSsT/EAsF)

[7. Flachner B, Kovári Z, Varga A, Gugolya Z, Vonderviszt F, Náray-Szabó G, et al. Role of phosphate chain mobility of MgATP in completing the 3-phosphoglycerate kinase catalytic site: binding, kinetic, and crystallographic studies with ATP and MgATP. Biochemistry. 2004;43:3436–49.](http://paperpile.com/b/dMwSsT/Z0Ag)

[8. Gallois-Montbrun S, Faraj A, Seclaman E, Sommadossi J-P, Deville-Bonne D, Véron M. Broad specificity of human phosphoglycerate kinase for antiviral nucleoside analogs. Biochem. Pharmacol. 2004;68:1749–56.](http://paperpile.com/b/dMwSsT/dh6z)

[9. Model K. Hexokinase of Human Erythrocytes. Eur. J. Riochem. 1974;45:39–52.](http://paperpile.com/b/dMwSsT/fNy8)

[10. Brownson C, Spencer N. Kinetic studies on the two common inherited forms of human erythrocyte adenylate kinase. Biochem. J. 1972;130:805–11.](http://paperpile.com/b/dMwSsT/l88J)

[11. Soldin SJ, Balinsky D. Kinetic properties of human erythrocyte glucose 6-phosphate dehydrogenase. Biochemistry. 1968;7:1077–82.](http://paperpile.com/b/dMwSsT/xAn7)

[12. Adediran SA. Kinetic properties of normal human erythrocyte glucose-6-phosphate dehydrogenase dimers. Biochimie. 1991;73:1211–8.](http://paperpile.com/b/dMwSsT/bTD8)

[13.](http://paperpile.com/b/dMwSsT/UYDn) Wang CS, Alaupovic P. Glyceraldehyde-3-phosphate dehydrogenase from human erythrocyte membranes. Kinetic mechanism and competitive substrate inhibition by glyceraldehyde 3-phosphate. Archives of biochemistry and biophysics. 1980 Nov 30;205(1):136-45.

[14. Orsi BA, Cleland WW. Inhibition and kinetic mechanism of rabbit muscle glyceraldehyde-3-phosphate dehydrogenase. Biochemistry. 1972;11:102–9.](http://paperpile.com/b/dMwSsT/fGVn)

[15.](http://paperpile.com/b/dMwSsT/mFm6) Eby D, Kirtley ME. Isolation and characterization of glyceraldehyde-3-phosphate dehydrogenase from human erythrocyte membranes. Archives of biochemistry and biophysics. 1979 Dec 31;198(2):608-13.

[16. Otto M, Heinrich R, Kühn B, Jacobasch G. A Mathematical Model for the Influence of Fructose 6-Phosphate, ATP, Potassium, Ammonium and Magnesium on the Phosphofructokinase from Rat Erythrocytes. Eur. J. Biochem. Blackwell Publishing Ltd; 1974;49:169–78.](http://paperpile.com/b/dMwSsT/EsUh)

[17.](http://paperpile.com/b/dMwSsT/9CVv) Hanson RL, Rudolph FB, Lardy HA. Rabbit Muscle Phosphofructokinase THE KINETIC MECHANISM OF ACTION AND THE EQUILIBRIUM CONSTANT. Journal of Biological Chemistry. 1973 Nov 25;248(22):7852-9.

[18. Otto M, Heinrich R, Jacobasch G, Rapoport S. A mathematical model for the influence of anionic effectors on the phosphofructokinase from rat erythrocytes. Eur. J. Biochem. 1977;74:413–20.](http://paperpile.com/b/dMwSsT/GosQ)

[19. Holzhütter HG, Jacobasch G, Bisdorff A. Mathematical modelling of metabolic pathways affected by an enzyme deficiency. A mathematical model of glycolysis in normal and pyruvate-kinase-deficient red blood cells. Eur. J. Biochem. 1985;149:101–11.](http://paperpile.com/b/dMwSsT/pJzv)

[20. Hall ER, Cottam GL. Isozymes of pyruvate kinase in vertebrates: their physical, chemical, kinetic and immunological properties. Int. J. Biochem. 1978;9:785–93.](http://paperpile.com/b/dMwSsT/fSQq)

[21.](http://paperpile.com/b/dMwSsT/0RtE) Wang CS. Inhibition of human erythrocyte lactate dehydrogenase by high concentrations of pyruvate. European Journal of Biochemistry. 1977 Sep 1;78(2):569-74.

[22. Mohrenweiser HW, Novotny JE. An enzymatically inactive variant of human lactate dehydrogenase-LDHBGUA-1 Study of subunit interaction. Biochimica et Biophysica Acta (BBA) - Protein Structure and Molecular Enzymology. 1982;702:90–8.](http://paperpile.com/b/dMwSsT/HDTb)

[23. Thorburn DR, Kuchel PW. Regulation of the human-erythrocyte hexose-monophosphate shunt under conditions of oxidative stress. A study using NMR spectroscopy, a kinetic isotope effect, a reconstituted system and computer simulation. Eur. J. Biochem. 1985;150:371–86.](http://paperpile.com/b/dMwSsT/Y6n7)

[24. Topham CM, Matthews B, Dalziel K. Kinetic studies of 6-phosphogluconate dehydrogenase from sheep liver. Eur. J. Biochem. 1986;156:555–67.](http://paperpile.com/b/dMwSsT/u9pK)

[25. Villet RH, Dalziel K. The nature of the carbon dioxide substrate and equilibrium constant of the 6-phosphogluconate dehydrogenase reaction. Biochem. J. 1969;115:633–8.](http://paperpile.com/b/dMwSsT/EB9N)

[26. Plesser T, Wurster B, Hess B. Determination of the Kinetic Constants of Glucosephosphate Isomerase by Non-linear Optimization. Eur. J. Biochem. Blackwell Publishing Ltd; 1979;98:93–8.](http://paperpile.com/b/dMwSsT/Nt7U)
